# Supplementary material for: Systematic profiling of conditional degron tag technologies for target validation studies
Source: Nat Commun. 2022 Sep 20;13:5495. doi: 10.1038/s41467-022-33246-4 (PMC9489723; doi:10.1038/s41467-022-33246-4)

Systematic profiling of conditional degron tag technologies for target validation studies

Daniel P Bondeson[1], Zachary Mullin-Bernstein[1], Sydney Oliver[1], Thomas A Skipper[1], Thomas C Atack[1], Nolan Bick[1], Meilani Ching[1], Andrew A Guirguis[1,2,3,4], Jason Kwon[1,2], Carly Langan[1], Dylan Millson[1,2], Brenton R Paolella[1], Kevin Tran[3,4], Sarah J Wie[1], Francisca Vazquez[1], Zuzana Tothova[1,2], Todd R Golub[1,2], William R Sellers[1,2], Alessandra Ianari[1,5,6]

Supplementary Information

Supplementary Figure 1 - Justification of doses used in this study, related to Figure 1

Supplementary Figure 2 - Immunoblot assessments of degradation and expression, related to Figure 2

Supplementary Figure 3 - Immunoblot assessments of degradation and expression, related to Figure 2 (continued)

Supplementary Figure 4 - Immunoblot assessments of degradation and expression, related to Figure 2 (continued)

Supplementary Figure 5 - Immunoblot assessments of degradation and expression, related to Figure 2 (continued)

Supplementary Figure 6 - Immunoblot assessments of degradation and expression, related to Figure 2 (continued)

Supplementary Figure 7 - POI-CDT expression levels are variable, and high expression can prevent degradation, related to Figure 2

Supplementary Figure 8 - Comparison of AID and AID2 systems across 9 targets

Supplementary Figure 9 - SMASh degron fusions have unique kinetics of degradation relative to other degron technologies, related to Figure 3

Supplementary Figure 10 - SMASh degron fusions have unique kinetics of degradation relative to other degron technologies, related to Figure 3 (continued)

Supplementary Figure 11 - CDTs activity is largely independent of cellular context with few exceptions, related to Figure 4

Supplementary Figure 12 - CDTs have target-specific effects on functional activity, related to Figure 4

Supplementary Table 1 - Published pharmacokinetic data for degrader drugs

Supplementary Note 1 - Standard Operating Protocol for the Generation of Functionally Relevant and Degradable CDT-Target Alleles

Supplementary Note 2 - Synthesis of dTAG^{v}-1

Supplementary References

Source Data - Blot data for Supplementary Figures

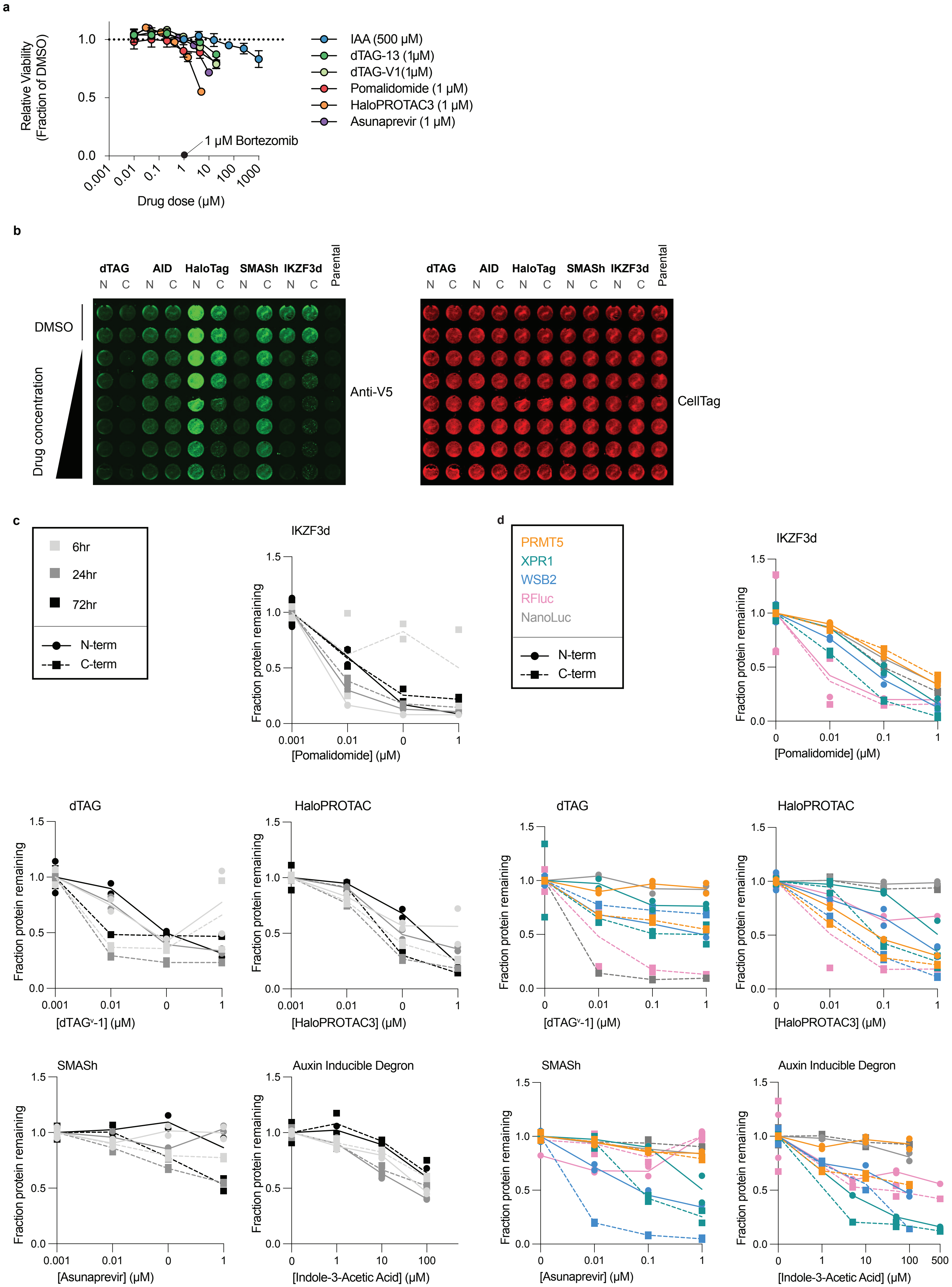
a
Relative Viability (Fraction of DMSO)
1.0
0.5
0.0
0.001
0.01
0.1
1
10
100
1000
Drug dose (µM)
1 µM Bortezomib
IAA (500 µM)
dTAG-13 (1µM)
dTAG-V1(1µM)
Pomalidomide (1 µM)
HaloPROTAC3 (1 µM)
Asunaprevir (1 µM)
b
dTAG AID HaloTag SMASh IKZF3d Parental
N C N C N C N C N C
DMSO
Drug concentration
Anti-V5
CellTag
c
6hr
24hr
72hr
N-term
C-term
IKZF3d
Fraction protein remaining
[Pomalidomide] (µM)
dTAG
[dTAGᵛ-1] (µM)
HaloPROTAC
[HaloPROTAC3] (µM)
SMASh
[Asunaprevir] (µM)
Auxin Inducible Degron
[Indole-3-Acetic Acid] (µM)
d
PRMT5
XPR1
WSB2
RFluc
NanoLuc
N-term
C-term
IKZF3d
dTAG
HaloPROTAC
SMASh
Auxin Inducible Degron
500

Supplementary Figure 1 - Justification of doses used in this study, related to Figure 1

a) Viability assays in HEK-293T after treatment with degron drugs at relevant concentrations. Cells were treated for 72 hours prior to viability assessment by Cell Titer Glo. Bortezomib, the cytotoxic proteasome inhibitor, is used as a positive control for cell death. To the right, the maximal concentrations of each drug in degradation assays is indicated for reference. Error bars represent the mean and standard error of N=3 technical replicates and are representative of N=2 independent experiments.
b) In-cell western assay to evaluate degradation of CDT fusions. 293T expressing the indicated PGK-NanoLuc-CDT proteins were plated in 96w plates and treated with the corresponding drug under the indicated conditions. Cells were then fixed, permeabilized, and stained with anti-V5 antibody (green, left) and co-stained with CellTag (red, right). Shown is a representative image for 24 hours of drug treatment.
c) Quantification of NanoLuc in-cell western assays across doses and timepoints. Anti-V5 intensity was quantified for each well and normalized to total cellular protein using CellTag.
d) Quantification of the extent of degradation across different doses for the indicated proteins using ICW.

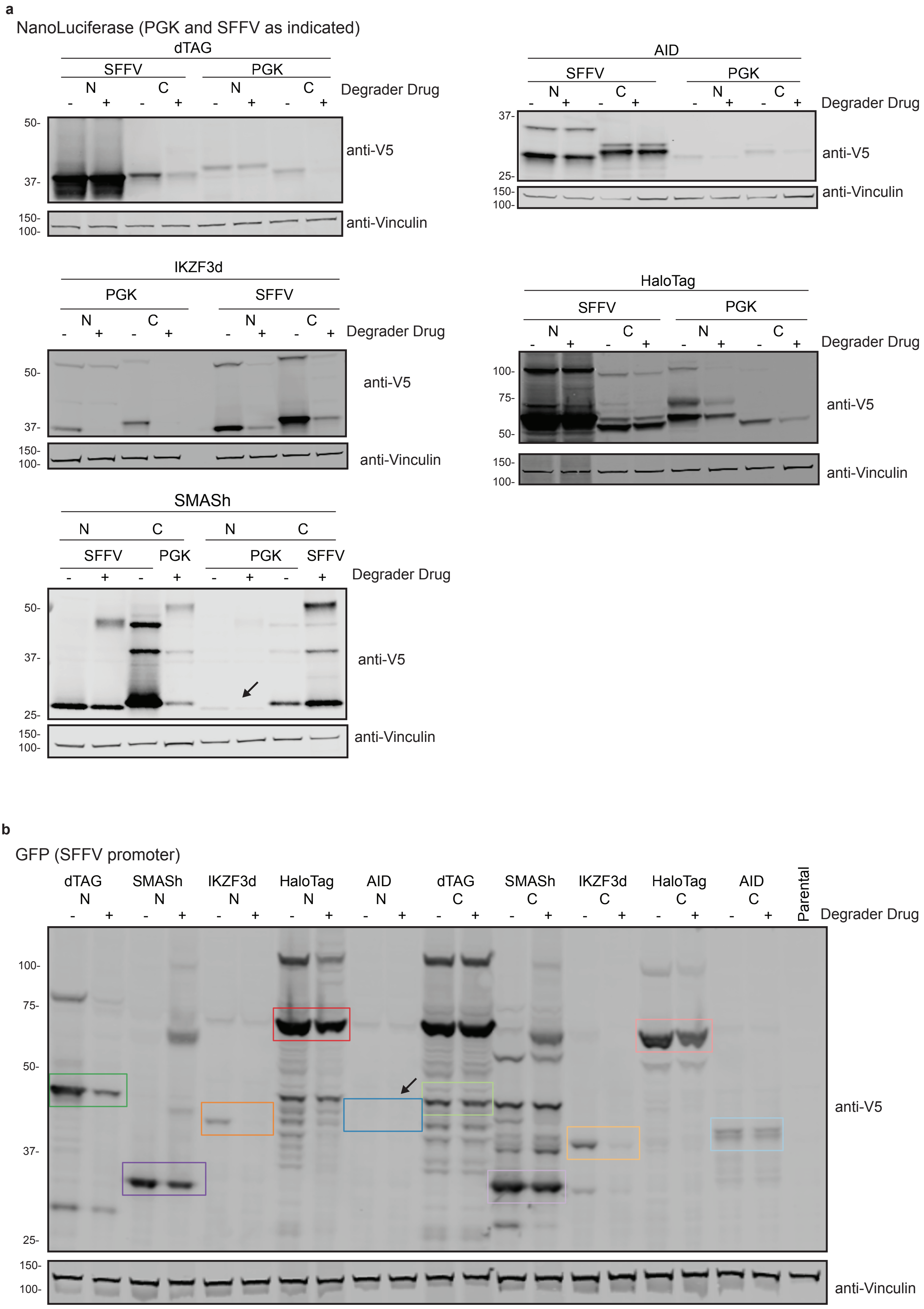
a
NanoLuciferase (PGK and SFFV as indicated)
dTAG
SFFV
PGK
N
C
Degrader Drug
anti-V5
anti-Vinculin
50-
37-
150-
100-
AID
25-
IKZF3d
HaloTag
75-
SMASh
b
GFP (SFFV promoter)
dTAG
SMASh
IKZF3d
HaloTag
AID
Parental

Supplementary Figure 2 - Immunoblot assessments of degradation and expression, related to Figure 2

a) Cells stably expressing the indicated conditional degron tag (CDT) fusions to NanoLuciferase were treated for 24 hours with degrader drugs, and fusion protein abundance was assessed by anti-V5 immunoblot. Vinculin is a house-keeping protein used for normalization. The following concentrations of degrader drug were used for each technology: AID (100 or 500 μM IAA), dTAG (1 μM dTAG13 or 1 μM dTAG^{V}-1), IKZF3d (1 μM Pomalidomide), HaloTag (1 μM HaloPROTAC3), SMASh (1 μM Asunaprevir). Data for this panel and all panels evaluating degradation performance for a target across the entire CDT panel are representative of N=2 independent experiments,
b) Same as in **a**, but with GFP CDT fusion proteins.

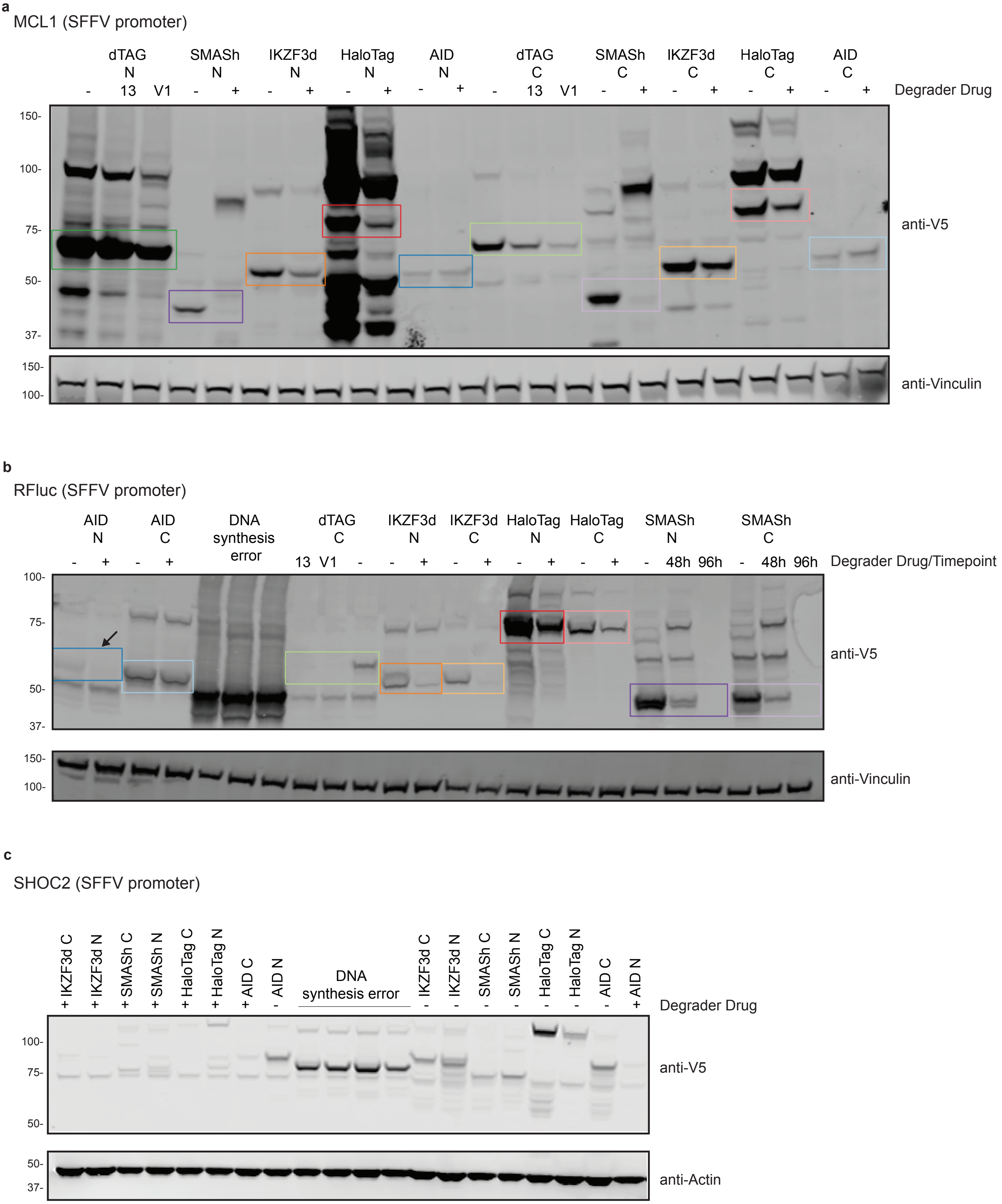
a
MCL1 (SFFV promoter)
dTAG N
SMASh N
IKZF3d N
HaloTag N
AID N
dTAG C
SMASh C
IKZF3d C
HaloTag C
AID C
- 13 V1 - + - + - + - + - 13 V1 - + - + - + - +
Degrader Drug
150-
100-
75-
50-
37-
anti-V5
150-
100-
anti-Vinculin
b
RFluc (SFFV promoter)
AID N
AID C
DNA synthesis error
dTAG C
IKZF3d N
IKZF3d C
HaloTag N
HaloTag C
SMASh N
SMASh C
- + - + 13 V1 - - + - + - + - + - 48h 96h - 48h 96h
Degrader Drug/Timepoint
100-
75-
50-
37-
anti-V5
150-
100-
anti-Vinculin
c
SHOC2 (SFFV promoter)
+ IKZF3d C
+ IKZF3d N
+ SMASh C
+ SMASh N
+ HaloTag C
+ HaloTag N
+ AID C
- AID N
DNA synthesis error
- IKZF3d C
- IKZF3d N
- SMASh C
- SMASh N
- HaloTag C
- HaloTag N
- AID C
+ AID N
Degrader Drug
100-
75-
50-
anti-V5
50-
37-
anti-Actin

Supplementary Figure 3 - Immunoblot assessments of degradation and expression, related to Figure 2 (continued)

a) Same as in Supplementary Figure 2**a**, but with MCL1 CDT fusion proteins.
b) Same as in Supplementary Figure 2**a**, but with RFluc CDT fusion proteins, and the following concentrations: AID (500 µM IAA), and SMASh (10 µM Asunaprevir, treated for 48 or 96 hours where indicated).
c) Same as in Supplementary Figure 2**a**, but with SHOC2 CDT fusion proteins.

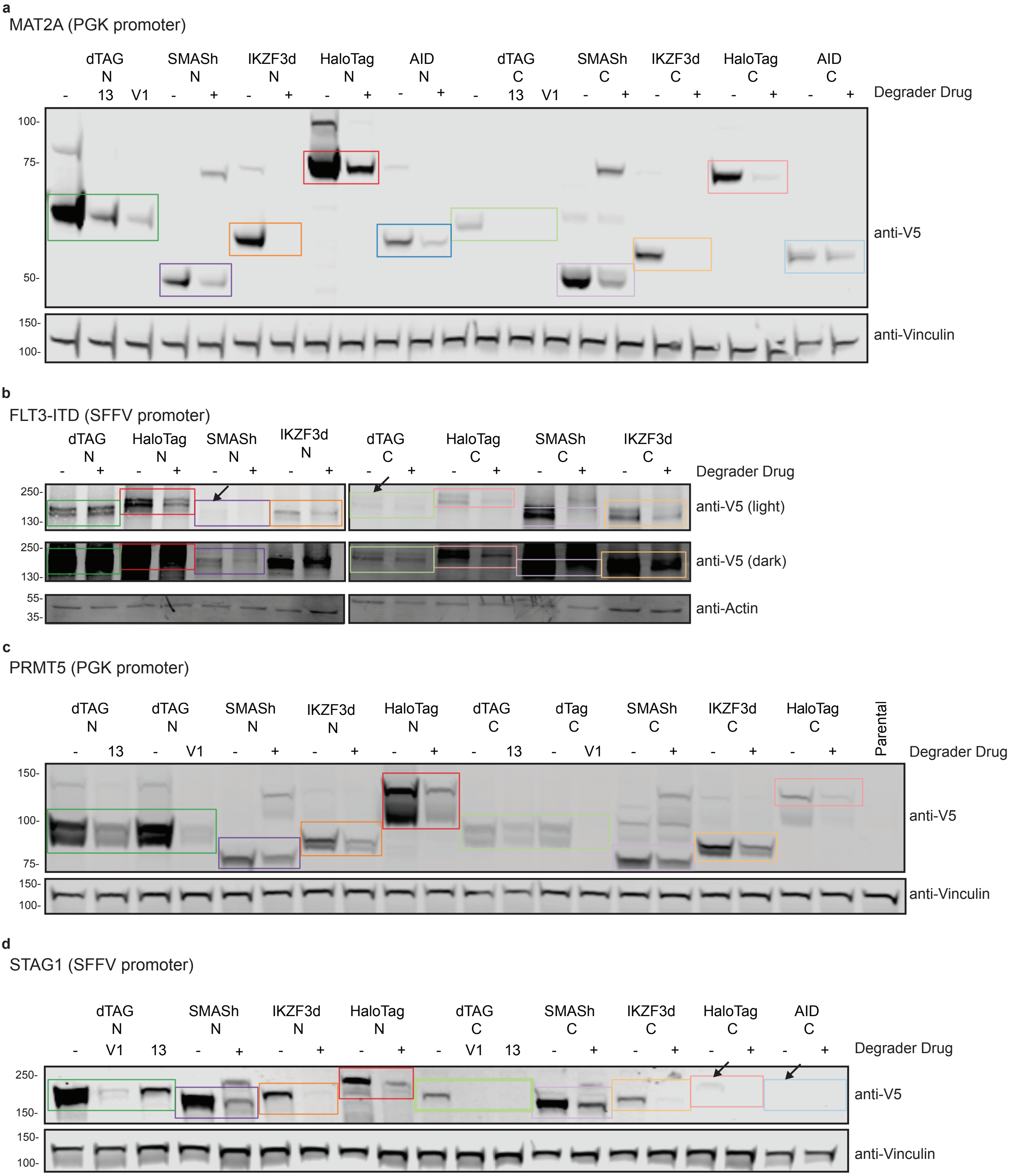
a
MAT2A (PGK promoter)
dTAG N
SMASh N
IKZF3d N
HaloTag N
AID N
dTAG C
SMASh C
IKZF3d C
HaloTag C
AID C
- 13 V1 - + - + - + - + - 13 V1 - + - + - + - +
Degrader Drug
100-
75-
50-
anti-V5
150-
100-
anti-Vinculin
b
FLT3-ITD (SFFV promoter)
dTAG N
HaloTag N
SMASh N
IKZF3d N
dTAG C
HaloTag C
SMASh C
IKZF3d C
- + - + - + - + - + - + - + - +
Degrader Drug
250-
130-
anti-V5 (light)
anti-V5 (dark)
55-
35-
anti-Actin
c
PRMT5 (PGK promoter)
dTAG N
dTAG N
SMASh N
IKZF3d N
HaloTag N
dTAG C
dTag C
SMASh C
IKZF3d C
HaloTag C
Parental
- 13 - V1 - + - + - + - 13 - V1 - + - + - +
Degrader Drug
150-
100-
75-
anti-V5
anti-Vinculin
d
STAG1 (SFFV promoter)
dTAG N
SMASh N
IKZF3d N
HaloTag N
dTAG C
SMASh C
IKZF3d C
HaloTag C
AID C
- V1 13 - + - + - + - V1 13 - + - + - + - +
Degrader Drug
250-
150-
100-
anti-V5
anti-Vinculin

Supplementary Figure 4 - Immunoblot assessments of degradation and expression, related to Figure 2 (continued)

a) Same as in Supplementary Figure 2**a**, but with MAT2A CDT fusion proteins.
b) Same as in Supplementary Figure 2**a**, but with FLT3-ITD CDT fusion proteins.
c) Same as in Supplementary Figure 2**a**, but with PRMT5 CDT fusion proteins.
d) Same as in Supplementary Figure 2**a**, but with STAG1 CDT fusion proteins.

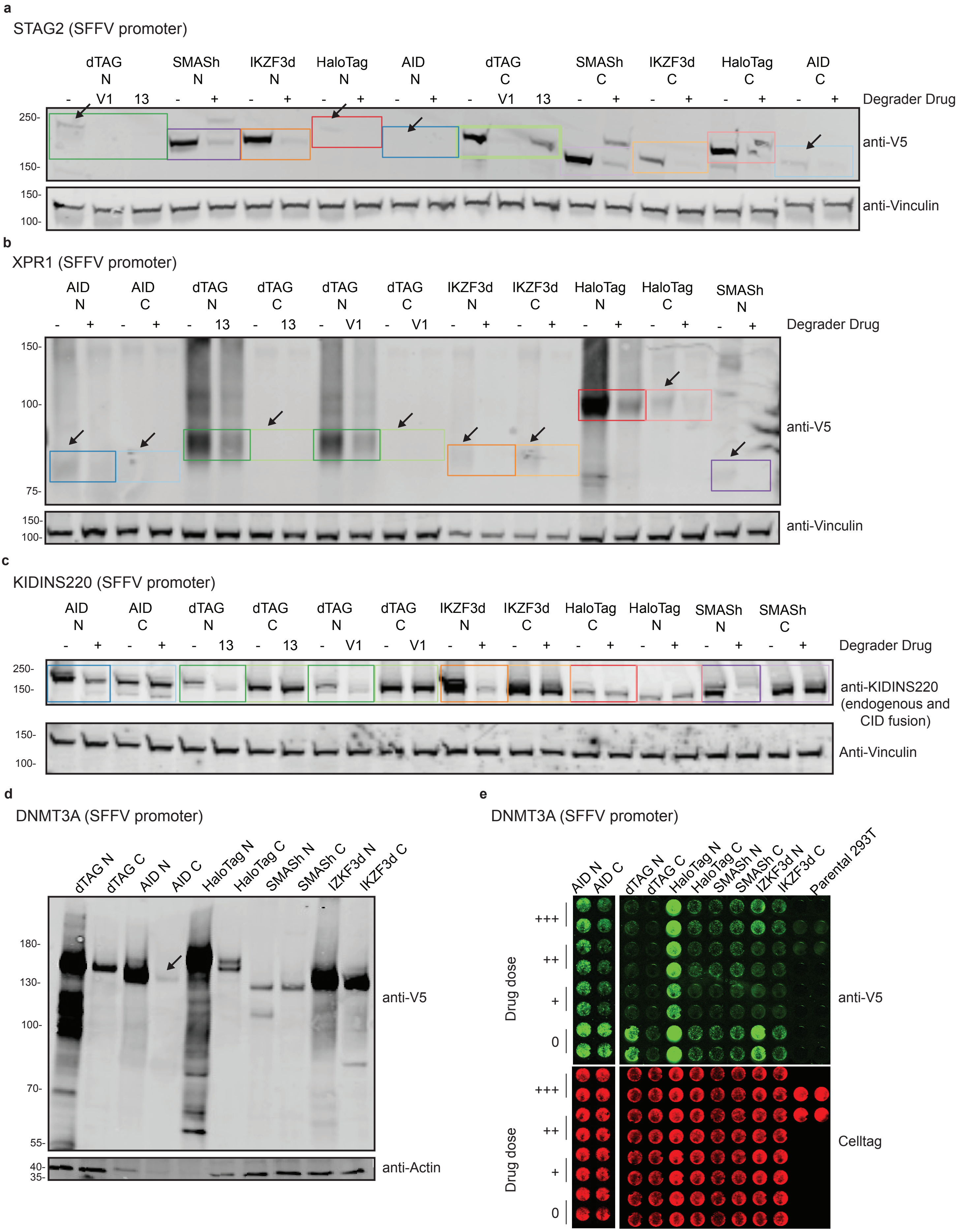
a
STAG2 (SFFV promoter)
dTAG N
SMASh N
IKZF3d N
HaloTag N
AID N
dTAG C
SMASh C
IKZF3d C
HaloTag C
AID C
- V1 13 - + - + - + - + - V1 13 - + - + - + - +
Degrader Drug
250-
150-
anti-V5
150-
100-
anti-Vinculin
b
XPR1 (SFFV promoter)
AID N
AID C
dTAG N
dTAG C
dTAG N
dTAG C
IKZF3d N
IKZF3d C
HaloTag N
HaloTag C
SMASh N
- + - + - 13 - 13 - V1 - V1 - + - + - + - + - +
Degrader Drug
150-
100-
75-
anti-V5
150-
100-
anti-Vinculin
c
KIDINS220 (SFFV promoter)
AID N
AID C
dTAG N
dTAG C
dTAG N
dTAG C
IKZF3d N
IKZF3d C
HaloTag C
HaloTag N
SMASh N
SMASh C
- + - + - 13 - 13 - V1 - V1 - + - + - + - + - + - +
Degrader Drug
250-
150-
anti-KIDINS220 (endogenous and CID fusion)
150-
100-
Anti-Vinculin
d
DNMT3A (SFFV promoter)
dTAG N
dTAG C
AID N
AID C
HaloTag N
HaloTag C
SMASh N
SMASh C
IZKF3d N
IKZF3d C
180-
130-
100-
70-
55-
anti-V5
40-
35-
anti-Actin
e
DNMT3A (SFFV promoter)
AID N
AID C
dTAG N
dTAG C
HaloTag N
HaloTag C
SMASh N
SMASh C
IZKF3d N
IKZF3d C
Parental 293T
Drug dose
+++
++
+
0
anti-V5
Drug dose
+++
++
+
0
Celltag

Supplementary Figure 5 - Immunoblot assessments of degradation and expression, related to Figure 2 (continued)

a) Same as in Supplementary Figure 2**a**, but with STAG2 CDT fusion proteins.
b) Same as in Supplementary Figure 2**a**, but with XPR1 CDT fusion proteins.
c) Same as in Supplementary Figure 2**a**, but with KIDINS220 CDT fusion proteins.
d) Same as in Supplementary Figure 2**a**, but with DNMT3A CDT fusions without treatment with the respective degrader drug
e) In-cell western to assess degradation of DNMT3A CDT fusion proteins. Note that expression levels were quantified using the traditional western blot (**n**), but that the extent of degradation was calculated from the in-cell western. Drug concentrations used were 10 nM, 100 nM, and 1 μM for all drugs except for IAA, which was used at 5 μM, 50 μM, and 500 μM.

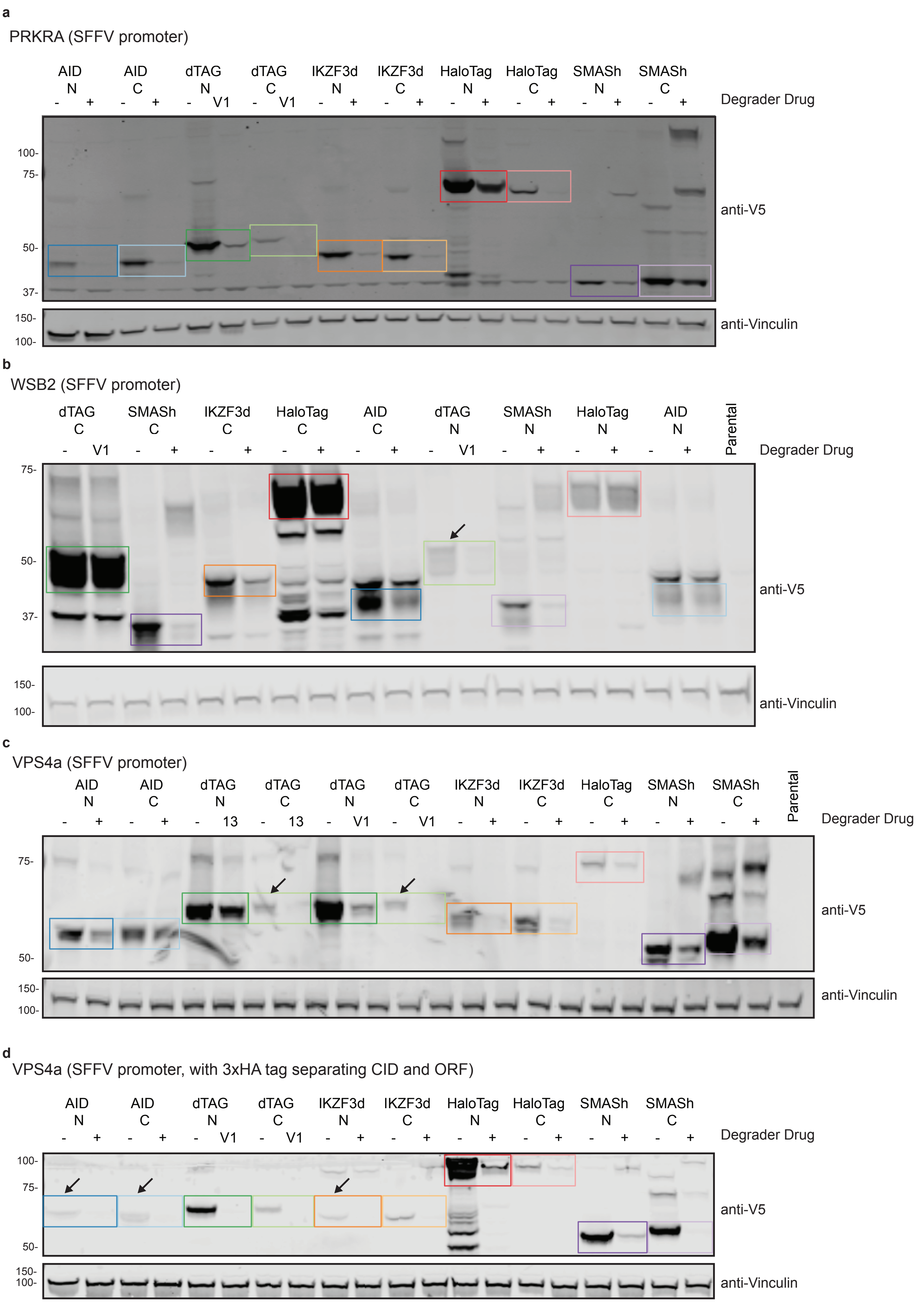
a
PRKRA (SFFV promoter)
AID N
AID C
dTAG N
dTAG C
IKZF3d N
IKZF3d C
HaloTag N
HaloTag C
SMASh N
SMASh C
- + - + - V1 - V1 - + - + - + - + - + - +
Degrader Drug
100-
75-
50-
37-
anti-V5
150-
100-
anti-Vinculin
b
WSB2 (SFFV promoter)
dTAG C
SMASh C
IKZF3d C
HaloTag C
AID C
dTAG N
SMASh N
HaloTag N
AID N
Parental
- V1 - + - + - + - + - V1 - + - + - +
Degrader Drug
75-
50-
37-
anti-V5
150-
100-
anti-Vinculin
c
VPS4a (SFFV promoter)
AID N
AID C
dTAG N
dTAG C
dTAG N
dTAG C
IKZF3d N
IKZF3d C
HaloTag C
SMASh N
SMASh C
Parental
- + - + - 13 - 13 - V1 - V1 - + - + - + - + - +
Degrader Drug
75-
50-
anti-V5
150-
100-
anti-Vinculin
d
VPS4a (SFFV promoter, with 3xHA tag separating CID and ORF)
AID N
AID C
dTAG N
dTAG C
IKZF3d N
IKZF3d C
HaloTag N
HaloTag C
SMASh N
SMASh C
- + - + - V1 - V1 - + - + - + - + - + - +
Degrader Drug
100-
75-
50-
anti-V5
150-
100-
anti-Vinculin

Supplementary Figure 6 - Immunoblot assessments of degradation and expression, related to Figure 2 (continued)

a) Same as in Supplementary Figure 2**a**, but with PRKRA degron fusions.
b) Same as in Supplementary Figure 2**a**, but with WSB2 degron fusions.
c) Same as in Supplementary Figure 2**a**, but with VPS4A CDT fusion proteins using the standard panel design.
d) Same as in Supplementary Figure 2**a**, but with VPS4A CDT fusion proteins incorporating a 3x-HA linker in between the CDT and the VPS4A protein.

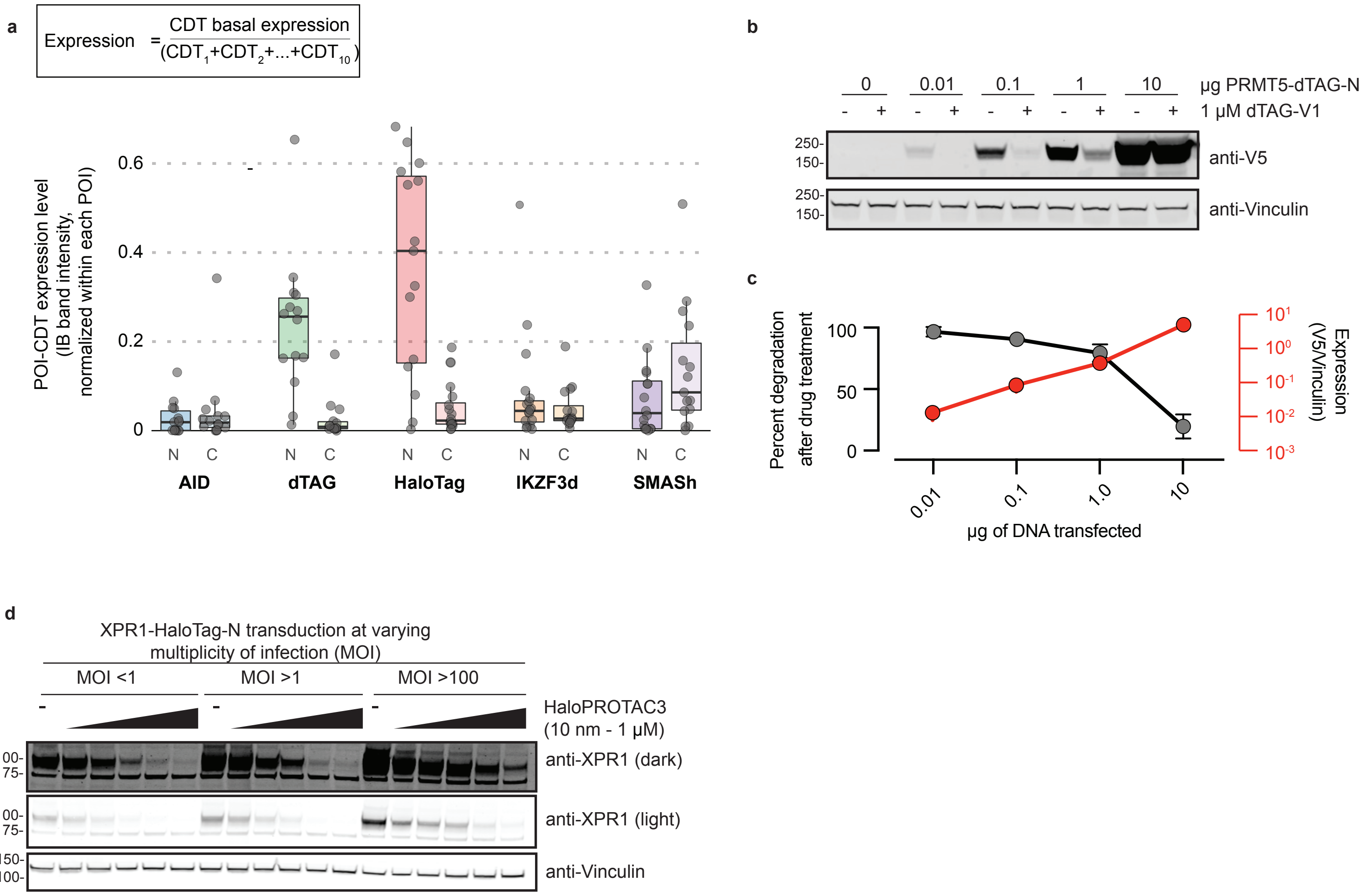

a
Expression = CDT basal expression / (CDT1+CDT2+...+CDT10)
POI-CDT expression level (IB band intensity, normalized within each POI)
0.6
0.4
0.2
0
N C N C N C N C N C
AID
dTAG
HaloTag
IKZF3d
SMASh
b
0 0.01 0.1 1 10 µg PRMT5-dTAG-N
- + - + - + - + - + 1 µM dTAG-V1
250-
150-
anti-V5
anti-Vinculin
c
Percent degradation after drug treatment
100
50
0
Expression (V5/Vinculin)
10^1
10^0
10^-1
10^-2
10^-3
0.01 0.1 1.0 10
µg of DNA transfected
d
XPR1-HaloTag-N transduction at varying multiplicity of infection (MOI)
MOI <1
MOI >1
MOI >100
HaloPROTAC3 (10 nm - 1 µM)
100-
75-
anti-XPR1 (dark)
anti-XPR1 (light)
150-
100-
anti-Vinculin

Supplementary Figure 7 - POI-CDT expression levels are variable, and high expression can prevent degradation, related to Figure 2

a) Expression levels across each degron technology. The anti-V5 IB signal of the expected molecular weight was normalized to a loading control (typically Vinculin or beta-Actin) and is plotted as a fraction of the total V5 signal from that particular target as in Figure 1c. Thus, differences within a target between different CDT fusion proteins is evident, but differences across different targets cannot be drawn. The box and whisker plot indicates the median, the 1st and 3rd quartiles, and 1.5x the interquartile range. N=16 targets analyzed for each construct except dTAG-N, where N=15.
b) Comparison of degradation potency for the same target expressed at different levels. The indicated amounts of a plasmid encoding PRMT-dTAG-N expressed from a PGK promoter was transiently transfected into 293T cells, and cells were subsequently treated with dTAG^{V}-1 for 24 hours before analysis by IB.
c) Quantification of panel **b**. The baseline anti-V5 expression signal (red dots, right axis) and the percent reduction in total protein levels (grey dots, left axis). Error bars represent the mean and standard error between two independent experiments.
d) Comparison of degradation potency for XPR1-HaloTag-N degron fusions at various expression levels. The cells described in **d** were treated with the indicated doses of HaloPROTAC3 for 72 hours before analysis by IB. Data are representative of N=3 experiments using the same cell lines.

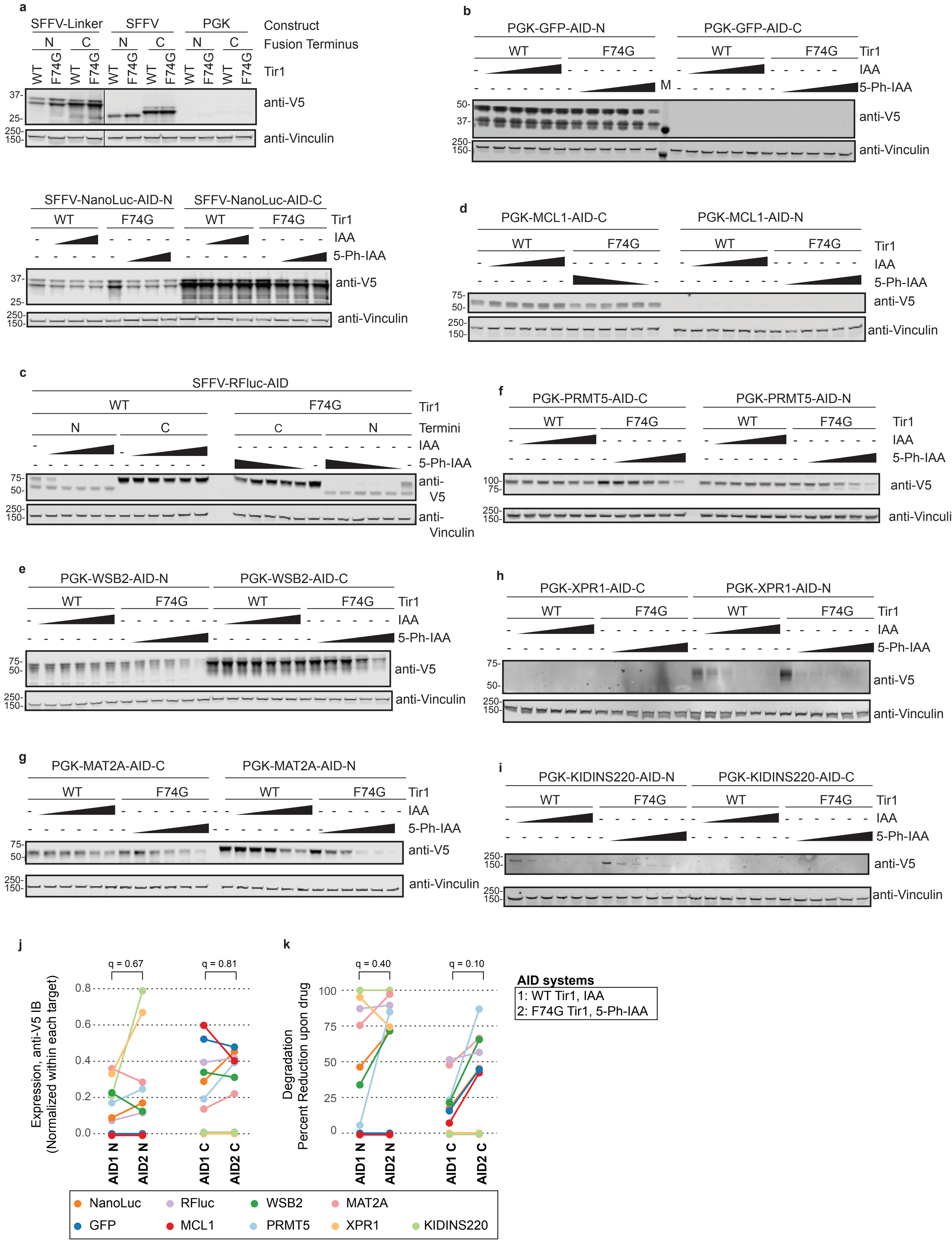
a
SFFV-Linker SFFV PGK Construct
N C N C N C Fusion Terminus
WT F74G WT F74G WT F74G WT F74G WT F74G WT F74G Tir1
anti-V5
anti-Vinculin
b
PGK-GFP-AID-N PGK-GFP-AID-C
WT F74G WT F74G Tir1
IAA
M 5-Ph-IAA
anti-V5
anti-Vinculin
SFFV-NanoLuc-AID-N SFFV-NanoLuc-AID-C
WT F74G WT F74G Tir1
IAA
5-Ph-IAA
anti-V5
anti-Vinculin
d
PGK-MCL1-AID-C PGK-MCL1-AID-N
WT F74G WT F74G Tir1
IAA
5-Ph-IAA
anti-V5
anti-Vinculin
c
SFFV-RFluc-AID
WT F74G Tir1
N C C N Termini
IAA
5-Ph-IAA
anti-V5
anti-Vinculin
f
PGK-PRMT5-AID-C PGK-PRMT5-AID-N
WT F74G WT F74G Tir1
IAA
5-Ph-IAA
anti-V5
anti-Vinculi
e
PGK-WSB2-AID-N PGK-WSB2-AID-C
WT F74G WT F74G Tir1
IAA
5-Ph-IAA
anti-V5
anti-Vinculin
h
PGK-XPR1-AID-C PGK-XPR1-AID-N
WT F74G WT F74G Tir1
IAA
5-Ph-IAA
anti-V5
anti-Vinculin
g
PGK-MAT2A-AID-C PGK-MAT2A-AID-N
WT F74G WT F74G Tir1
IAA
5-Ph-IAA
anti-V5
anti-Vinculin
i
PGK-KIDINS220-AID-N PGK-KIDINS220-AID-C
WT F74G WT F74G Tir1
IAA
5-Ph-IAA
anti-V5
anti-Vinculin
j
Expression, anti-V5 IB (Normalized within each target)
q = 0.67 q = 0.81
AID1 N AID2 N AID1 C AID2 C
k
Degradation Percent Reduction upon drug
q = 0.40 q = 0.10
AID1 N AID2 N AID1 C AID2 C
AID systems
1: WT Tir1, IAA
2: F74G Tir1, 5-Ph-IAA
NanoLuc RFluc WSB2 MAT2A
GFP MCL1 PRMT5 XPR1 KIDINS220

Supplementary Figure 8 - Comparison of AID and AID2 systems across 9 targets

a) NanoLuc-AID constructs were expressed in HEK-293T cells co-expressing Tir1 (either wildtype (WT) for AID or the F74G variant for AID2). Top, expression of different NanoLuc constructs without drug treatment. The “SFFV-Linker” construct contains a rigid linker in between the NanoLuc and AID constructs, compared to the other constructs which contain no linker. Note that irrelevant lanes were cropped from this blot, but that the entire immunoblot was treated consistently. Bottom, Cells were treated for 24 hours with IAA for AID (at 100 nM, 1 µM, 10 µM, or 100 µM) or 5-Ph-IAA for AID2 (at 100 nM, 1µM, 10 µM, or 100 µM) and then NanoLuc-AID expression levels were evaluated by immunoblot. Data are representative of N=2 independent experiments, as are all panels evaluating the AID and AID2 systems.
b) Same as in **a**, but for GFP-AID constructs treated with IAA (100 nM, 1 µM, 10 µM, 100 µM, or 500 µM) or 5-Ph-IAA (100 nM, 1 µM, 10 µM, 100 µM, or 500 µM). M, marker.
c) Same as in **b**, but for RFluc-AID constructs.
d) Same as in **b**, but for MCL1-AID constructs.
e) Same as in **b**, but for WSB2-AID constructs.
f) Same as in **b**, but for PRMT5-AID constructs.
g) Same as in **b**, but for MAT2A-AID constructs.
h) Same as in **b**, but for XPR1-AID constructs.
i) Same as in **b**, but for KIDINS220-AID constructs.
j) Comparison of expression levels between AID and AID2 systems. The baseline expression of each target was quantified from immunoblot (panels a-i) and normalized by the average immunoblot of the 4 constructs for that target (AID and AID2, N and C-termini). Lines connect the points corresponding to each target to enable comparison of AID and AID2 systems. Q-values indicate the result of a t-test comparing the expression levels between the AID and AID2 systems for both N- and C-terminal tags, adjusted for multiple comparisons using Holm’s method (N-terminus N = 9, df = 11.3, statistic = -1.00; C-terminus N = 9, df = 15.7, statistic = -0.24)
k) Same as in **i**, but comparing degradation efficiency. (N-terminus: N = 9, df = 15.9, statistic = -0.85; C-terminus: N = 9, df = 13.6, statistic = -2.2)

a

	RFluc	NanoLuc	VPS4a	PRMT5	WSB2	MAT2A	GFP	XPR1	KIDINS220
AID	N	N	N	C	N	N			
dTAG	C	C	N	N	N	N	N	C	N
IKZF3d	C	N	N	N	N	C	C	N	N
HaloTag		C	C	C	N	N	C	N	N
SMASh		C	C	N	N	N		C	

b

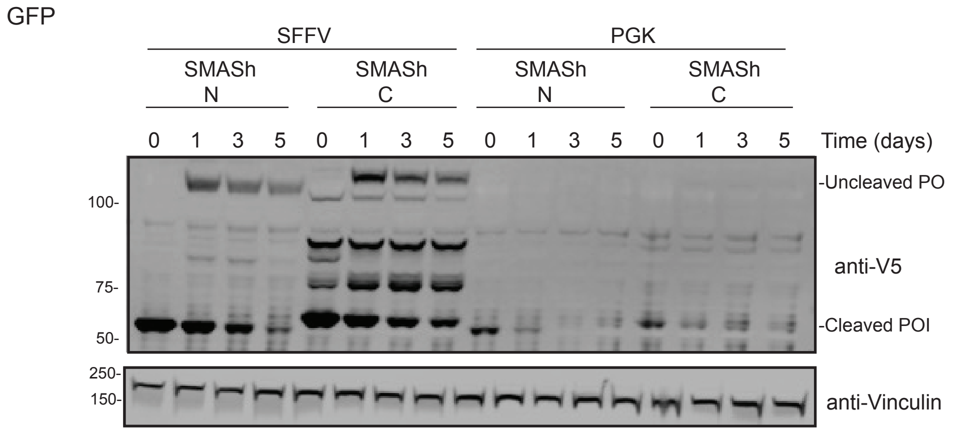

c

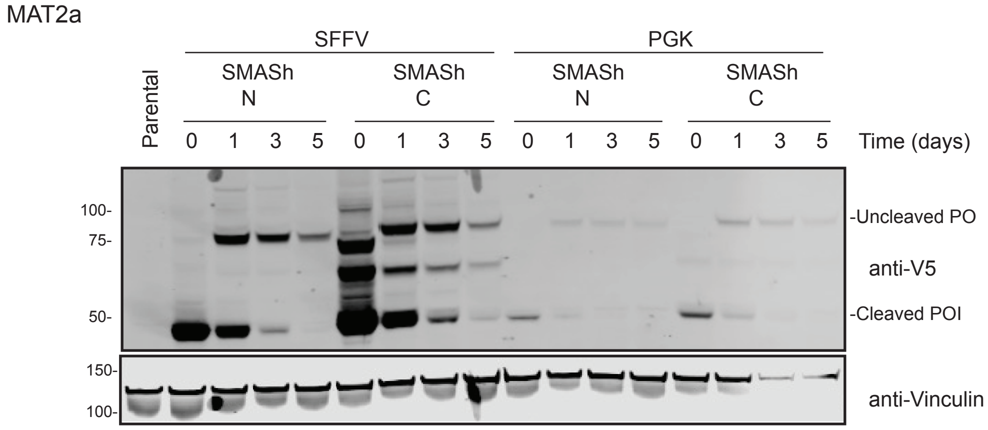

d

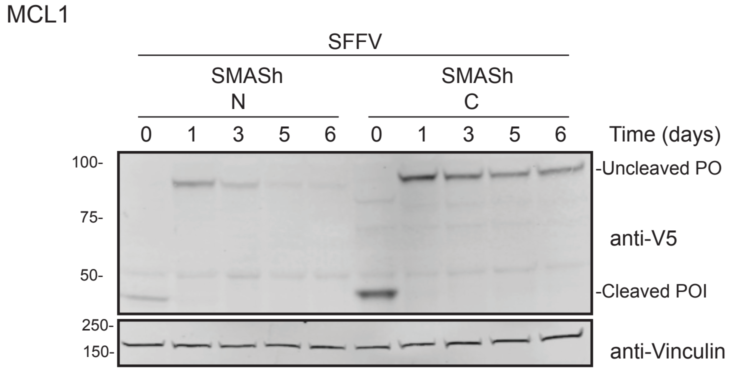

Supplementary Figure 9 - SMASh degron fusions have unique kinetics of degradation relative to other degron technologies, related to Figure 3

a) Table indicating which degron fusion protein was profiled in Figure 3.
b) 293T cells stably expressing the indicated GFP degron fusion protein were treated for various times before protein analysis by IB. Labels indicate expected molecular weight of the cleaved “tagless” protein of interest and the uncleaved tagged form. Experiments evaluating kinetics of degradation for SMASh proteins were repeated at least N=2 times, and representative blots are shown.
c) Same as in **b**, but for MAT2a.
d) Same as in **b**, but for MCL1.

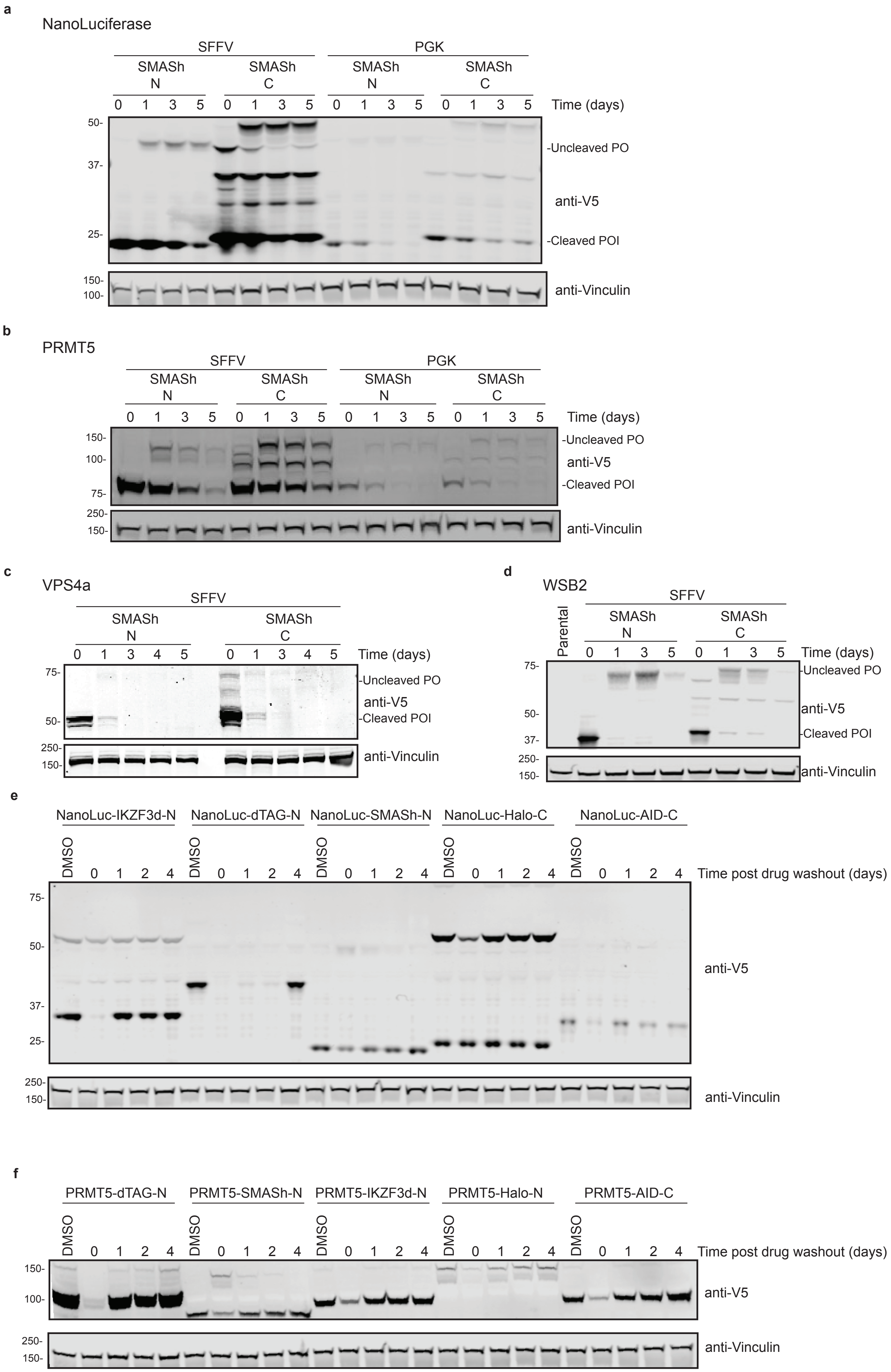
a
NanoLuciferase
SFFV
PGK
SMASh N
SMASh C
SMASh N
SMASh C
0 1 3 5 0 1 3 5 0 1 3 5 0 1 3 5
Time (days)
50-
37-
25-
-Uncleaved PO
anti-V5
-Cleaved POI
150-
100-
anti-Vinculin
b
PRMT5
SFFV
PGK
SMASh N
SMASh C
SMASh N
SMASh C
0 1 3 5 0 1 3 5 0 1 3 5 0 1 3 5
Time (days)
150-
100-
75-
-Uncleaved PO
anti-V5
-Cleaved POI
250-
150-
anti-Vinculin
c
VPS4a
SFFV
SMASh N
SMASh C
0 1 3 4 5 0 1 3 4 5
Time (days)
75-
50-
-Uncleaved PO
anti-V5
-Cleaved POI
250-
150-
anti-Vinculin
d
WSB2
SFFV
Parental
SMASh N
SMASh C
0 1 3 5 0 1 3 5
Time (days)
75-
50-
37-
-Uncleaved PO
anti-V5
-Cleaved POI
250-
150-
anti-Vinculin
e
NanoLuc-IKZF3d-N
NanoLuc-dTAG-N
NanoLuc-SMASh-N
NanoLuc-Halo-C
NanoLuc-AID-C
DMSO 0 1 2 4 DMSO 0 1 2 4 DMSO 0 1 2 4 DMSO 0 1 2 4 DMSO 0 1 2 4
Time post drug washout (days)
75-
50-
37-
25-
anti-V5
250-
150-
anti-Vinculin
f
PRMT5-dTAG-N
PRMT5-SMASh-N
PRMT5-IKZF3d-N
PRMT5-Halo-N
PRMT5-AID-C
DMSO 0 1 2 4 DMSO 0 1 2 4 DMSO 0 1 2 4 DMSO 0 1 2 4 DMSO 0 1 2 4
Time post drug washout (days)
150-
100-
anti-V5
250-
150-
anti-Vinculin

Supplementary Figure 10 - SMASh degron fusions have unique kinetics of degradation relative to other degron technologies, related to Figure 3 (continued)

a) Same as in Supplementary Figure 9**b**, but for NanoLuciferase.
b) Same as in Supplementary Figure 9**b b**, but for PRMT5.
c) Same as in Supplementary Figure 9**b b**, but for VPS4a.
d) Same as in Supplementary Figure 9**b b**, but for WSB2.
e) Washout analysis of NanoLuc-CDT fusions to assess resynthesis after drug removal. Cells expressing the indicated CDT fusions were pre-treated with the respective degrader drug prior to removing the drug-containing medium and incubating the cells for the indicated periods of time with medium without drug. Protein levels were then assessed by IB.
f) Same as in **e** but with PRTM5-CDT fusion proteins.

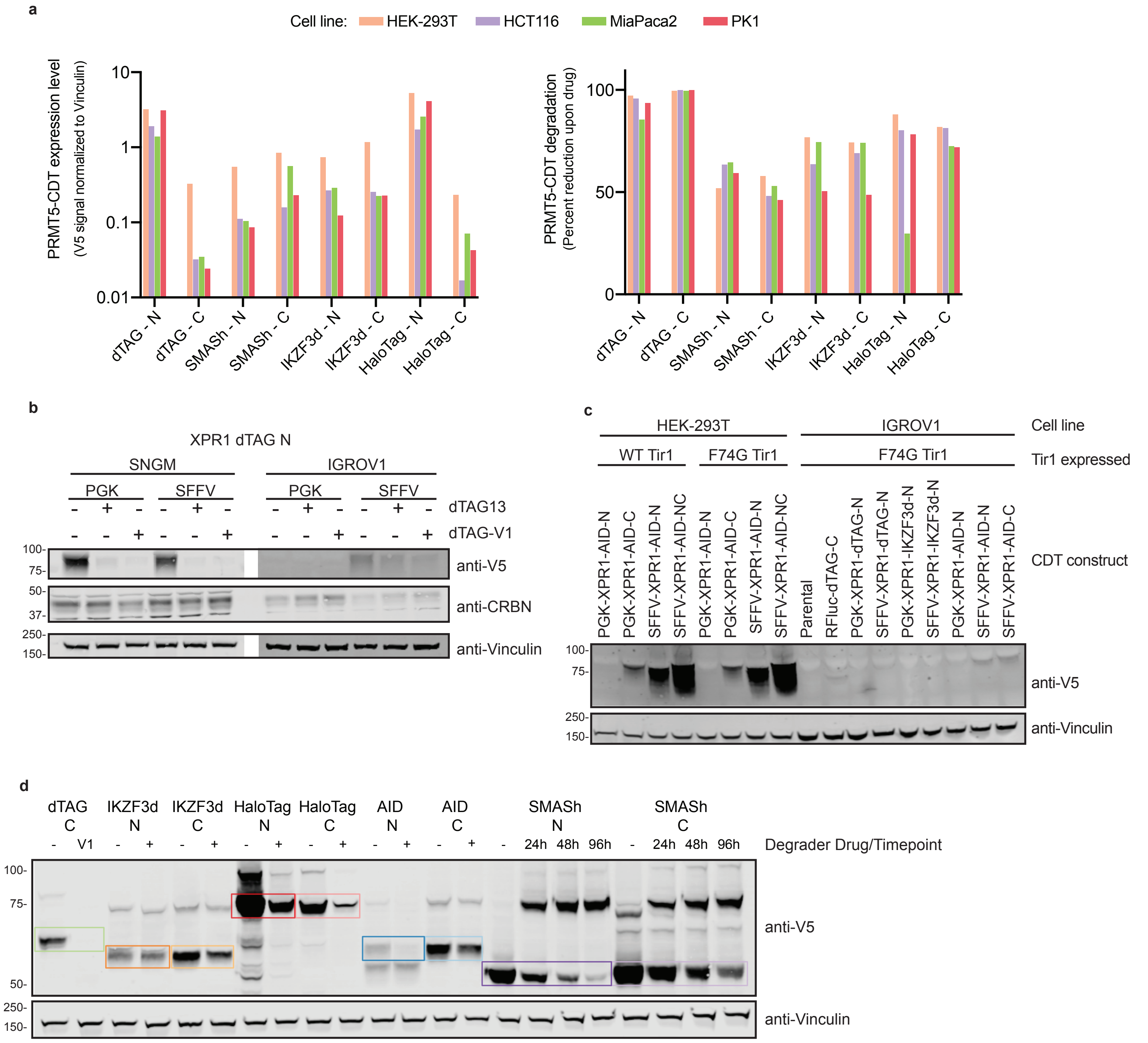
a
Cell line: HEK-293T HCT116 MiaPaca2 PK1
PRMT5-CDT expression level (V5 signal normalized to Vinculin)
10
1
0.1
0.01
dTAG - N
dTAG - C
SMASh - N
SMASh - C
IKZF3d - N
IKZF3d - C
HaloTag - N
HaloTag - C
PRMT5-CDT degradation (Percent reduction upon drug)
100
50
0
b
XPR1 dTAG N
SNGM
IGROV1
PGK
SFFV
dTAG13
dTAG-V1
100-
75-
50-
37-
250-
150-
anti-V5
anti-CRBN
anti-Vinculin
c
HEK-293T
IGROV1
Cell line
WT Tir1
F74G Tir1
Tir1 expressed
PGK-XPR1-AID-N
PGK-XPR1-AID-C
SFFV-XPR1-AID-N
SFFV-XPR1-AID-NC
Parental
RFluc-dTAG-C
PGK-XPR1-dTAG-N
SFFV-XPR1-dTAG-N
PGK-XPR1-IKZF3d-N
SFFV-XPR1-IKZF3d-N
SFFV-XPR1-AID-C
CDT construct
anti-V5
anti-Vinculin
d
dTAG C
IKZF3d N
IKZF3d C
HaloTag N
HaloTag C
AID N
AID C
SMASh N
SMASh C
V1
24h 48h 96h
Degrader Drug/Timepoint
anti-V5
anti-Vinculin

Supplementary Figure 11 - CDTs activity is largely independent of cellular context with few exceptions, related to Figure 4

a) Expression and degradation comparison of SFFV-expressed PRMT5 degron fusions in four different cell lines.
b) Comparison of degradation efficiency for XPR1 dTAG-N fusion proteins in uterine cancer cell lines (SNGM) and ovarian cancer cell lines (IGROV1). Stably expressing cell lines were treated for 72 hours with the indicated drug before immunoblot analysis. The anti-XPR1 immunoblot was analyzed separately as the signal from the SNGM cell line was too strong to detect the IGROV1 signal. IGROV1 cells were engineered via lentiviral transduction N=2 times, and one representative immunoblot is shown.
c) Comparison of expression levels for XPR1-AID fusion proteins when expressed in HEK-293T cells or IGROV1 cells. Stably expressed protein was evaluated by immunoblot. IGROV1 cells were engineered via lentiviral transduction N=2 times, and one representative immunoblot is shown.
d) Evaluation of RFluc-CDT expression and degradation in NIH-3T3 cells, as compared to Supplementary Figure 3d. The degradation performance was assessed N=2 times, and one representative immunoblot is shown.

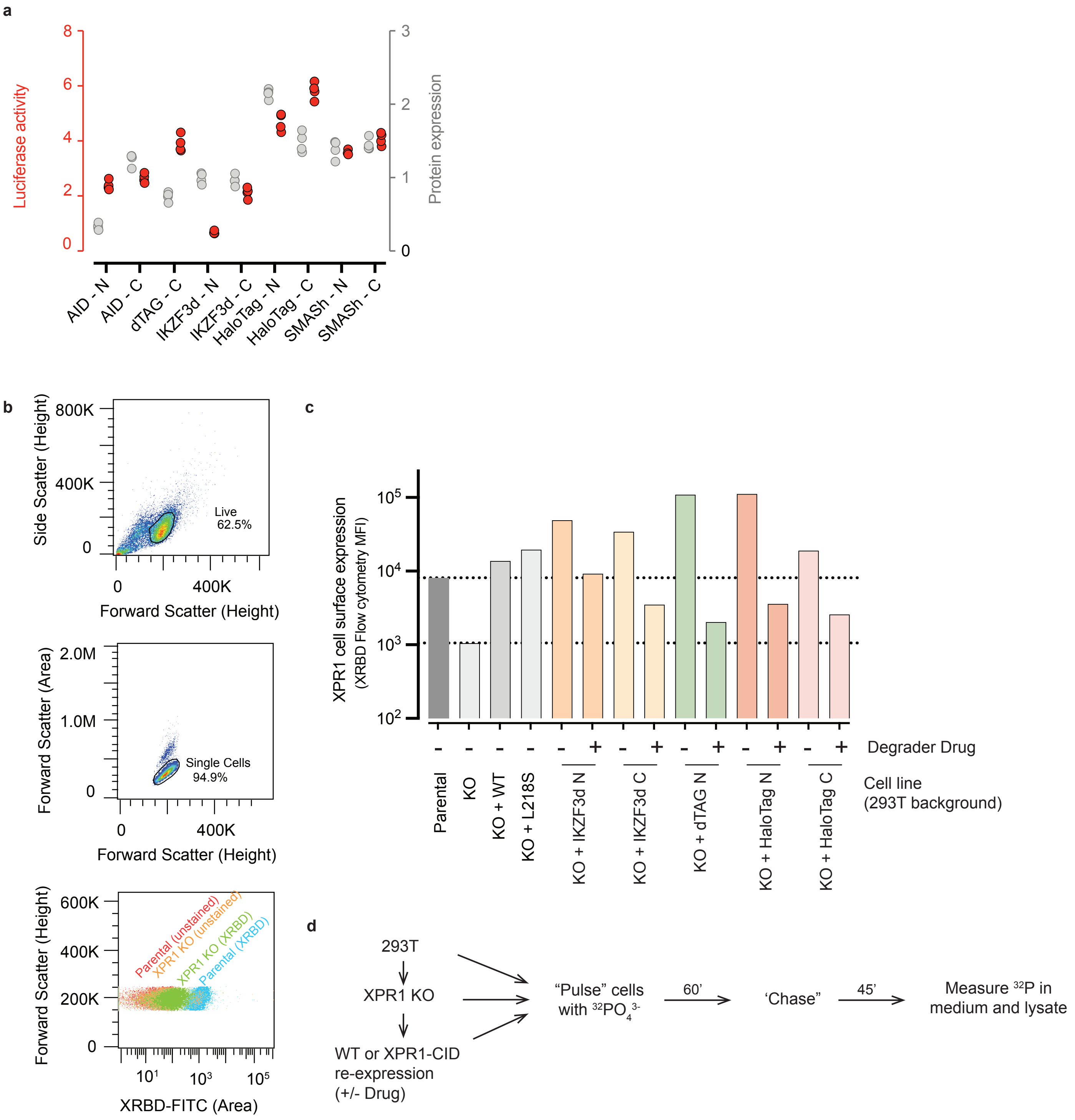

a
Luciferase activity
Protein expression
AID - N
AID - C
dTAG - C
IKZF3d - N
IKZF3d - C
HaloTag - N
HaloTag - C
SMASh - N
SMASh - C
b
Side Scatter (Height)
Forward Scatter (Height)
Live
62.5%
Forward Scatter (Area)
Single Cells
94.9%
Parental (unstained)
XPR1 KO (unstained)
XPR1 KO (XRBD)
Parental (XRBD)
XRBD-FITC (Area)
c
XPR1 cell surface expression
(XRBD Flow cytometry MFI)
Degrader Drug
Cell line
(293T background)
Parental
KO
KO + WT
KO + L218S
KO + IKZF3d N
KO + IKZF3d C
KO + dTAG N
KO + HaloTag N
KO + HaloTag C
d
293T
XPR1 KO
WT or XPR1-CID
re-expression
(+/- Drug)
"Pulse" cells
with $^{32}PO_4^{3-}$
60'
'Chase"
45'
Measure ^{32}P in
medium and lysate

Supplementary Figure 12 - CDTs have target-specific effects on functional activity, related to Figure 4

a) Luciferase activity assays for RFluc-CDT fusion proteins. On the left axis, the luciferase activity from a given well is divided by the total V5 in-cell western signal. The right axis is the V5 signal normalized to total cellularity as quantified by the CellTag stain.
b) Exemplar flow cytometry plots for evaluating XPR1 cell surface expression. Cell surface expression of XPR1 proteins was assessed by labeling cells with a receptor binding domain (XRBD) protein from xenotropic murine leukemia virus (NZB) which use XPR1 as an entry receptor (see main text and materials and methods). XRBD is detected with a FITC-conjugated secondary antibody. Top, gating to remove cellular debris. Middle, gating to isolate single cell populations. Bottom, FITC intensity across the indicated cell staining conditions.
c) Three days after addition of the degrader drug (1 µM Pomalidomide, 1 µM dTAG^{V}-1, or 1 µM HaloPROTAC3), XPR1 cell surface expression was assessed as in **d.** The Median Fluorescent Intensity of at least 10,000 cells is reported on the X-Axis.
d) Experimental plan for *XPR1* inactivation, re-expression of *XPR1* alleles (either untagged or as CDT fusions) and assessment of phosphate efflux.

Supplementary Table 1 - Published pharmacokinetic data for degrader drugs

Drug, ref, molecular weight (g/mol)		Dose administered	Time to max plasma concentration	Half-life ($T_{1/2}$)	Max plasma concentration	Area Under the Curve (AUC)	Clearance	Fraction Free
		mg/kg	hours	hours	ng/mL	hr*ng/mL	ml/min*kg	%
dTAG-V1[1] 1361.58	IV	2	0.08	3.02	7780	3200	10.1	NR
	IP	2	1.67	3.64	595	2200	10.7	NR
	IP	10	2	4.43	2123	18100	9.05	NR
	PO	NR	NR	NR	NR	NR	NR	NR
dTAG-13[1] 1049.18	IV	2	0.08	1.6	2370	1240	1250	32.5
	IP	10	2	2.41	1260	5620	6140	28
	PO	NR	NR	NR	NR	NR	NR	NR
Pomalidomide[2] 273.24	IV	5	NR	2.44	NR	7020	12.3	NR
	IP	NR	NR	NR	NR	NR	NR	NR
	PO	50	2	NR	3370	33100	NR	47.4
Asunaprevir[3] 748.29	IV	2	NR	4.6	NR	576000	57.3	NR
	IP	NR	NR	NR	NR	NR	NR	NR
	PO	5	6	NR	97300	404000	NA	28

IV, intravenous injection; IP, intraperitoneal injection; PO, oral delivery; NR, not reported

Supplementary Note 1 - Standard Operating Protocol for the Generation of Functionally Relevant and Degradable CDT-Target Alleles

CLONING

We have worked successfully with several CROs to have our inserts cloned in the whole panel (Twist Biosciences, Epoch Lifesciences and Genscript). Inserts can also be cloned in house, with restriction enzymes (BamH1-EcoR1) We recommend utilizing inserts with silent point mutations disrupting PAM sites of interest, to facilitate the downstream generation of KO-rescue cell lines using CRISPR/Cas9. Inserts do not need start or stop codons as these are included in the vector backbone. All constructs and vector maps are available on Addgene (#185760-185779). The available vectors contain GFP-CDT fusion; the GFP insert can be excised via EcoR1/BamH1 digestion.

LENTIVIRAL PREP AND GENERATION OF STABLE 293T CELL LINES

Viral prep and infections are performed in 293T cells seeded in 24 well plates to facilitate the parallel analysis of the entire vector panel (20 constructs).

LENTIVIRAL PREP

Day 1: Plate 100,000 293T cells in 0.5 mL in each well of a 24-well plate.

Day 2: Transfection
a) Add 250 ng of each degron plasmid to separate tubes.
b) Prepare a master mix of TransIT-LT1 (1.5 µL per transfection) and serum free Opti-MEM (50 µL per reaction). Incubate at RT while mixing the other plasmids.
c) Prepare a master mix of packaging (psPAX2, 250 ng per transfection reaction) and envelope (e.g. VSV-G, 25 ng per transfection reaction) in serum free Opti-MEM (50 µL per transfection)
d) Add 50 µL of the packaging/envelope master mix to each tube containing degron plasmid
e) Add 50 µL of the TransIT-LT1 master mix to each tube
f) Incubate the mixture for 30 minutes at room temperature.
g) Carefully add the final 100 µL mixture dropwise to cells. Be careful to avoid disrupting or detaching the cells

Day 3: Replace with 400 µL of fresh medium (DMEM+10% FBS + P/S/G)

Day 4: Collect the virus-containing medium and store it at 4°C. Refresh the cells with 400 µL fresh media to continue producing virus.

Day 5: Collect the remaining medium and pool with the medium collected on Day 4. Briefly centrifuge (1000g for 5 minutes) to remove cellular debris and filter using a syringe and a 0.4 µm filter. Proceed to infection on the same day if possible. Store remaining viral supernatant at -80°C in 180 µL.

STABLE CELL LINE GENERATION

Day 1: Infection with previously generated lentivirus
a. Plate HEK-293T cells at 100,000 cells in 500 µL per well in a 24-well plate. If required, use 293T-TIR1 (WT-Tir1 for AID1, F74G for AID2), parental 293T for all others constructs, and plate two extra wells as “No Infection Controls”
b. Add polybrene at a final concentration of 8 µg/mL

c. Add 200 µL of lentivirus to each well
d. Centrifuge the plates at 931g at 30ºC for 2 hours.

Day 2: Aspirate media and replace with fresh DMEM + 10% FBS + P/S/G.

Day 3: Add 2 µg/mL puromycin to all wells except one of the No Infection Controls.

Day 5 (or 6): No surviving cells should be observed in the non-infected control, indicating that puromycin selection is complete. At this point, evaluate the infection efficiency by comparing the number of cells in the non-infected control cells that were not treated with puromycin. We aim for 30-50% infection efficiency to ensure single integrations and enable accurate comparisons in expression levels. Subculture cell lines according to standard tissue culture practices.

INITIAL WESTERN BLOT (24 hours, 1 dose) TO TEST FOR CDT-TARGET FUSIONS EXPRESSION AND DEGRADATION

We observed a high variability of the basal expression and degradation of CDT-target fusions on a target by target basis. In some cases, no product can be detected at all. This step allows to deprioritize poorly expressed, or not degraded, targets and to bring forward a smaller set of constructs (4-6) for downstream kinetic and functionality experiments.

Day 1: Plate 293T-degron cells at 1,000,000 cells/well in 6-multiwell plates. Plate 2 wells for each construct: one for a DMSO control and a second for treatment with the respective degrader drug. In addition, plate one well of parental 293T cells as an immunoblot control.

Day 2: Treat cells with corresponding degrader compound. Treat all compounds at 1 µM, except AID for which the recommended top dose is 500 µM

Day 3: Collect cells for immunoblot according to standard practice (blotting with e.g. Cell Signaling V5-Tag (D3H8Q) Rabbit mAb #13202).

TIME COURSE AND DOSE RESPONSE

To quickly characterize CDT-target fusion kinetics of degradation we developed an In-Cell Western (ICW) protocol, as described below. We use three different concentrations of degrader (1 µM, 100 nm, 10 nm for dTAG-13/dTAG^{V}-1, Pomalidomide, HaloPROTAC3 and Asunaprenavir; 500 µM, 50 µM and 1 µM for Auxin/IAA) and at 3 time points (6, 24, 72 hours). These doses and timepoints are ideal for most constructs, but some CDT-fusion proteins may require longer timepoints, especially for SMASh-tag fusions.

Shown below is a representative example of an ICW plate map

Drug dose		1	2	3	4	5	6	7	8	9	10	11	12
DMSO	A	dTAG-N	dTAG-C	SMASh-N	SMASh-C	IKZF3d-N	IKZF3d-C	HaloTag-N	HaloTag-C	AID-N	AID-C	293T	
	B	dTAG-N	dTAG-C	SMASh-N	SMASh-C	IKZF3d-N	IKZF3d-C	HaloTag-N	HaloTag-C	AID-N	AID-C	293T	
10 nM / 5 µM	C	dTAG-N	dTAG-C	SMASh-N	SMASh-C	IKZF3d-N	IKZF3d-C	HaloTag-N	HaloTag-C	AID-N	AID-C	293T	
	D	dTAG-N	dTAG-C	SMASh-N	SMASh-C	IKZF3d-N	IKZF3d-C	HaloTag-N	HaloTag-C	AID-N	AID-C	293T	
100 nM / 50 µM	E	dTAG-N	dTAG-C	SMASh-N	SMASh-C	IKZF3d-N	IKZF3d-C	HaloTag-N	HaloTag-C	AID-N	AID-C	293T	
	F	dTAG-N	dTAG-C	SMASh-N	SMASh-C	IKZF3d-N	IKZF3d-C	HaloTag-N	HaloTag-C	AID-N	AID-C	293T	
1 µM / 500 µM	G	dTAG-N	dTAG-C	SMASh-N	SMASh-C	IKZF3d-N	IKZF3d-C	HaloTag-N	HaloTag-C	AID-N	AID-C	293T	
	H	dTAG-N	dTAG-C	SMASh-N	SMASh-C	IKZF3d-N	IKZF3d-C	HaloTag-N	HaloTag-C	AID-N	AID-C	293T	

Day 1: Plate stable cell lines in 96 well plate in 100 µL and grow overnight

a. Use 96-well, tissue-culture treated black plates with a clear bottom (e.g. VWR catalog number 89091-012). Avoid using white or clear plates due to their increased background.
b. Plate 40,000 cells per well for the 6 and 24 hour plates; plate 20,000 cells per well for the 72 hour plate.

Day 2: Cell treatment and 6 hour collection

a. Use any automated dispenser or manualling pipetting to treat cells according to layout above and return to the incubator overnight.
b. 6 hours after treatment, fix one plate according to the “Anti-V5 in-cell western protocol below.”

Day 3: 24 hours after treatment, fix one plate according to the “Anti-V5 in-cell western protocol below.”

Day 5: 72 hours after treatment, fix one plate according to the “Anti-V5 in-cell western protocol below.”

ANTI-V5 IN-CELL WESTERN PROTOCOL

This protocol was developed using a LI-COR Odyssey CLX instrument, but any system capable of scanning two colors from a 96-well plate should be suitable.

Assay components:

- Odyssey blocking buffer
- CellTag 700 Stain (LI-COR #926-41090)
- Cell Signaling V5-Tag (D3H8Q) Rabbit mAb #13202
- Anti-rabbit secondary antibody conjugated to IR-Dye 800CW (LI-COR #926-32211)
- 1X PBS
- Methanol
- 0.1% NP40 in PBS

1. Gently remove all medium from the wells. Manual pipetting, as opposed to vacuum aspiration, should be used to avoid lifting the cells.
2. Immediately fix cells by adding 100 µL of ice-cold methanol slowly to the side of each well to avoid disturbing the cells. Incubate at room temperature for 10 minutes with gentle shaking

3. Discard methanol using pipette
4. Wash five times with 200 µL of 0.1% NP40 in PBS for 5 minutes per wash. As the cells are fixed, buffer can be removed by inverting the plate and tapping it on a paper towel. Do not allow the cells to become dry.
5. Plates can be stored at 4ºC in PBS until all time-points are collected.
6. Once all plates have been fixed and washed, remove any remaining liquid by manual pipetting.
7. Gently add 100 µL of Odyssey blocking buffer to the cells and incubate for 30 minutes at room temperature with gentle shaking.
8. Dilute the V5 antibody 1:200 in Odyssey blocking buffer (you will need ~ 13.5 ml for all three plates)
9. Gently remove the blocking buffer and add 50 µL of diluted primary antibody to each well. Incubate overnight (~16 hours) at 4ºC with gentle shaking.
10. Remove the primary antibody and wash the plates five times with 200 µL of 0.1% NP40 in PBS. Each wash should be 5 minutes with gentle shaking.
11. Dilute secondary antibody (1:1000) + Cell Tag 700 (1:500) in Odyssey blocking buffer and add 50 µL to each well. Incubate for one hour at room temperature with gentle shaking, protecting the plates from light.
12. Wash plates 3 times with 200 µL 0.1% NP40 in PBS. Each should be 5 minutes with gentle shaking.

IMAGING - ODYSSEY

1. After final wash, completely remove any liquid from the wells. Prior to scanning, clean the bottom plate surface and the scanning bed with 70% Ethanol.
2. Scan plate with detection of both 700 and 800 channels. NOTE: adjust the height on the Odyssey and set it at 3mm.

Notes:

- Do not use plates with white walls - the autofluorescence will create background noise
- Protect the plates from light after addition of the IRdye secondary antibodies.
- Plates can be stored at 4ºC for 2-3 weeks after imaging.

WASHOUT ASSAY

Objective: Observe recovery kinetics of CDT-target fusion after treatment with degrader. We typically measure protein recovery at 24, 48, and 96hr post drug washout.

Day 1: Plate cells stably expressing degron tagged POI in three wells of a 6-well plate (DMSO, compound/no washout, compound + washout)

Day 2: Treat each cell line with the respective degrader drug.

Day 3: Collect DMSO and no washout controls. Store cell pellets at -20 until all samples are ready for immunoblot. Passage compound + washout cells in fresh media 1:2 for 24hr time point, 1:10 for 48hr time point, and 1:10 for 96hr time point

Day 4: Collect 24hr washout cells

Day 5: Collect 48hr washout cells and split 96hr 1:10 if necessary

Day 7: Collect 96hr washout and proceed to WB (probe with Cell Signaling V5-Tag (D3H8Q) Rabbit mAb #13202).

Supplemental Note 2 - Synthesis of dTAG-V1

This synthesis was based on a previously published method[4]

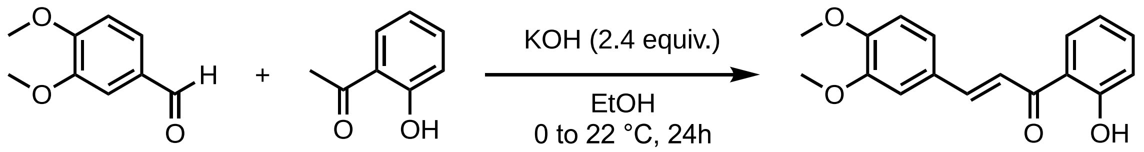

1 equiv. 1 equiv.

SI-1

(*E*)-3-(3,4-Dimethoxyphenyl)-1-(2-hydroxyphenyl)prop-2-en-1-one (SI-1). In a 100 mL round bottom flask, 3,4-dimethoxybenzaldehyde (2.01 g, 12.1 mmol, 1.0 equiv.) and 1-(2-hydroxyphenyl)ethan-1-one (1.5 mL, 12.1 mmol, 1.0 equiv.) were dissolved in 95% ethanol (24.3 mL). The reaction was cooled in an ice bath. KOH (1.65 g, 29.4 mmol, 2.4 equiv.) was added and the reaction was stirred at 0 °C for 30 minutes, after which the ice bath was removed and the reaction stirred for 24 hours at 22 °C. After 24 hours, the reaction was quenched by the addition 2N HCl until a yellow precipitate formed. The reaction was extracted with DCM, dried over $MgSO_4$, filtered, and concentrated by rotary evaporation. The product was crystallized from a mixture of hot ethanol and water to furnish **SI-1** (2.41 g, 8.48 mmol, 70% yield) as a yellow powder.

^{1}H NMR (400 MHz, CDCl3) δ 12.90 (s, 1H), 7.97 – 7.85 (m, 2H), 7.57 – 7.43 (m, 2H), 7.28 (m, 1H), 7.18 (d, *J* = 2.0 Hz, 1H), 7.03 (m, 1H), 7.00 – 6.89 (m, 2H), 3.97 (s, 3H), 3.95 (s, 3H).

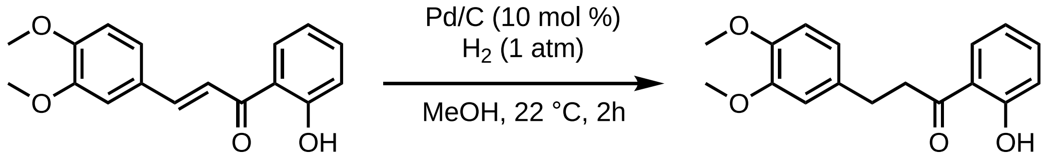

SI-1 **SI-2**

3-(3,4-Dimethoxyphenyl)-1-(2-hydroxyphenyl)propan-1-one) (SI-2). In a 50 mL round bottom flask with a stir bar, **SI-1** (1.0 g, 3.52 mmol, 1.0 equiv.) and Pd/C (10 mol %, 100 mg) were added and the flask capped with a rubber septum and purged twice with N_2. MeOH (17.6 mL) was added and the stir plate was set to 600 rpm. The nitrogen inlet was removed and a balloon of H_2 gas was affixed to the flask. An outlet needle was inserted into the septum and the flask was purged with H_2 for 10 minutes. The outlet needle was removed, a fresh balloon of H_2 was affixed, and the reaction was stirred at 22 °C until LCMS monitoring indicated the reaction was complete. Upon completion, the balloon was removed and the flask was flushed with N_2. The reaction was then filtered through silica and the filtrate washed with EtOAc. The crude filtrate was concentrated by rotary evaporation and purified by automated flash chromatography (RediSep Gold 40g, 0-100% EtOAc in hexanes) to furnish the **SI-2** (819.4 mg, 2.86 mmol, 81% yield) as an off-white solid.

^{1}H NMR (400 MHz, $CDCl_3$) δ 12.30 (s, 1H), 7.75 (m, 1H), 7.47 (m, 1H), 6.99 (m, 1H), 6.88 (m, 1H), 6.84 – 6.75 (m, 3H), 3.87 (d, *J* = 4.0 Hz, 6H), 3.31 (m, 2H), 3.03 (m, 2H).

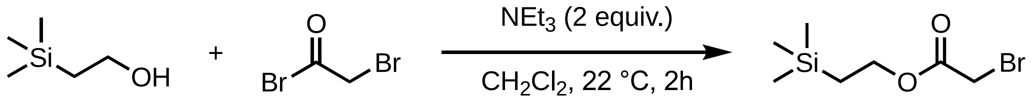

1 equiv. 1.2 equiv.

SI-3

2-(Trimethylsilyl)ethyl 2-bromoacetate (SI-3). In a 100 mL round bottom flask, 2-(trimethylsilyl)ethanol (1.2 mL, 8.46 mmol, 1.0 equiv.) was dissolved in DCM (33.8 mL). NEt_3 (2.4 mL, 16.9 mmol, 2.0 eq) was added followed by slow addition of bromoacetyl bromide (884 µL, 10.15 mmol, 1.2 eq). The reaction was stirred at 22 °C for 2 hours. The reaction was then diluted with EtOAc and washed with 2N HCl, water, and brine. The organic layer was dried over $MgSO_4$, filtered, and concentrated by rotary evaporation to furnish **SI-3** (1.91 g, 7.99 mmol, 94%) as a yellow oil which was used without further purification.

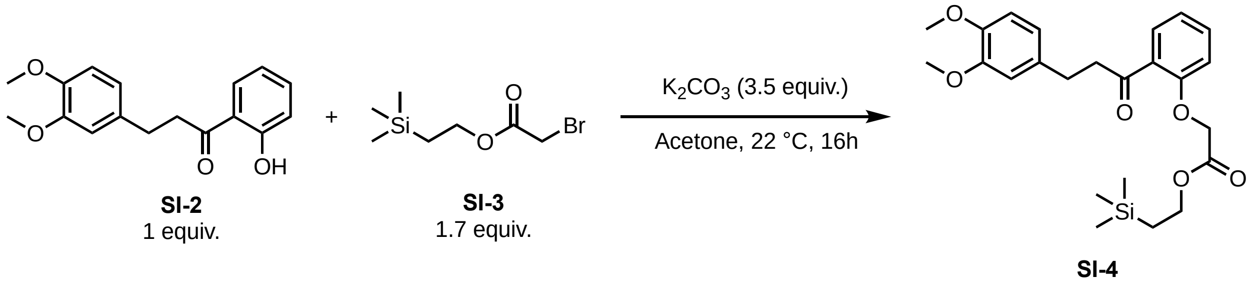

2-(Trimethylsilyl)ethyl 2-(2-(3-(3,4-dimethoxyphenyl)propanoyl)phenoxy)acetate (SI-4). In a 40 mL vial with a stir bar, **SI-2** (503.5 mg, 1.76 mmol, 1.0 equiv.), **SI-3** (715.1 mg, 2.99 mmol, 1.7 equiv.), and K_2CO_3 (850.7 mg, 6.16 mmol, 3.5 equiv.) were dissolved in acetone (8.8 mL) and stirred at 22 °C for 16 hours. The reaction was poured into a separatory funnel and diluted with EtOAc. The organic layer was washed successively with 2N HCl, water, and brine then dried over $MgSO_4$, filtered and concentrated. The crude mixture was purified by automated flash chromatography (RediSep Gold 40g, 0-50% EtOAc in hexanes) to furnish the **SI-4** (763.4 mg, 1.72 mmol, 98% yield) as a yellow oil.

^{1}H NMR (400 MHz, CDCl3) δ 7.72 – 7.65 (m, 1H), 7.47 – 7.38 (m, 1H), 7.09 – 7.00 (m, 1H), 6.85 – 6.73 (m, 4H), 4.67 (s, 2H), 4.32 – 4.23 (m, 2H), 3.85 (d, *J* = 7.3 Hz, 6H), 3.47 – 3.39 (m, 2H), 3.00 (t, *J* = 7.7 Hz, 2H), 1.03 – 0.94 (m, 2H), 0.04 (s, 9H).

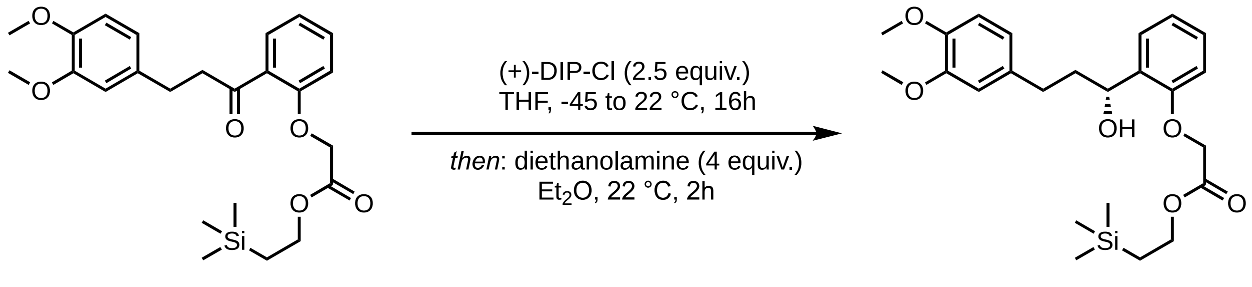

SI-4 **SI-5**

2-(Trimethylsilyl)ethyl (*R*)-2-(2-(3-(3,4-dimethoxyphenyl)-1-hydroxypropyl)phenoxy)acetate (SI-5). In a 40 mL vial, **SI-4** (500.0 mg, 1.13 mmol, 1.0 equiv.) was dissolved in THF (5.6 mL) and the vial was cooled to -45 °C in a dry ice/acetonitrile bath. (+)-*B*-Chlorodiisopinocampheylborane (1.6 M in hexane, 1.8 mL, 2.81 mmol, 2.5 equiv.) was added and the reaction was allowed to warm to 22 °C over 16 hours. The reaction was concentrated by rotary evaporation and dissolved in diethyl ether (22.5 mL). Diethanolamine (0.431 mL, 4.50 mmol, 4.0 equiv.) was added and the reaction was stirred at 22 °C for 2 hours. The reaction was filtered through a plug of silica and washed with EtOAc. The filtrate was concentrated and purified by automated flash chromatography (RediSep Gold 40g, 0-50% EtOAc in hexanes) to furnish **SI-5** (396.0 mg, 0.887 mmol, 78.8%,) as a colorless oil.

^{1}H NMR (400 MHz, $CDCl_3$) δ 7.33 – 7.28 (m, 1H), 7.25 – 7.19 (m, 1H), 7.03 – 6.96 (m, 1H), 6.81 – 6.74 (m, 4H), 4.97 – 4.84 (m, 1H), 4.72 – 4.60 (m, 2H), 4.34 – 4.25 (m, 2H), 3.86 (s,

3H), 3.85 (s, 3H), 3.31 (d, J = 6.5 Hz, 1H), 2.86 – 2.76 (m, 1H), 2.71 – 2.58 (m, 1H), 2.30 – 2.06 (m, 2H), 1.07 – 0.97 (m, 2H), 0.06 (s, 9H).

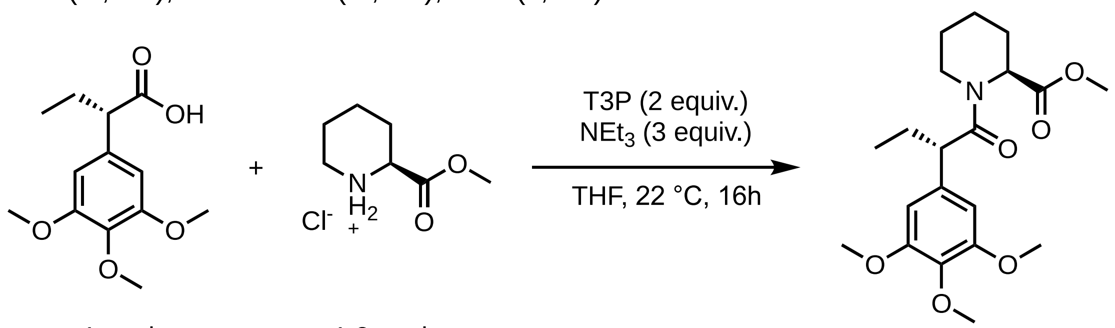

1 equiv. 1.2 equiv.

SI-6

Methyl (*S*)-1-((*S*)-2-(3,4,5-trimethoxyphenyl)butanoyl)piperidine-2-carboxylate (SI-6). In a 40 mL vial, (*S*)-2-(3,4,5-trimethoxyphenyl)butanoic acid (507.0 mg, 1.99 mmol, 1.0 equiv.), methyl (*S*)-piperidine-2-carboxylate hydrochloride (429.8 mg, 2.393 mmol, 1.200 equiv), and NEt_3 (1.0 mL, 5.98 mmol, 3.0 equiv.) were dissolved in THF (10.0 mL). T3P (50% w/w in EtOAc, 2.3 mL, 3.99 mmol, 2.0 equiv.) was added and the reaction stirred overnight. The reaction was diluted with EtOAc and extracted sequentially with 2N HCl, saturated $NaHCO_3$, and brine. The organic layer was dried over $MgSO_4$, filtered, and concentrated. The crude concentrate was purified by automated flash chromatography (RediSep Gold 40g, 0-100% EtOAc in hexanes) to furnish **SI-6** (681.5 mg, 1.80 mmol, 90% yield) as a white solid. NMR indicates a 3:1 mixture of amide rotamers.

^{1}H NMR (400 MHz, $CDCl_3$) δ 6.51 – 6.37 (m, 2H), 5.43 – 4.57 (m, 1H), 3.88 – 3.81 (m, 9H), 3.78 (s, 1H), 3.62 (s, 2H), 3.61 – 3.32 (m, 1H), 2.91 – 2.54 (m, 1H), 2.30 – 2.19 (m, 1H), 2.18 – 2.05 (m, 1H), 1.79 – 1.52 (m, 4H), 1.51 – 1.33 (m, 1H), 1.32 – 1.20 (m, 2H), 0.96 – 0.85 (m, 3H).

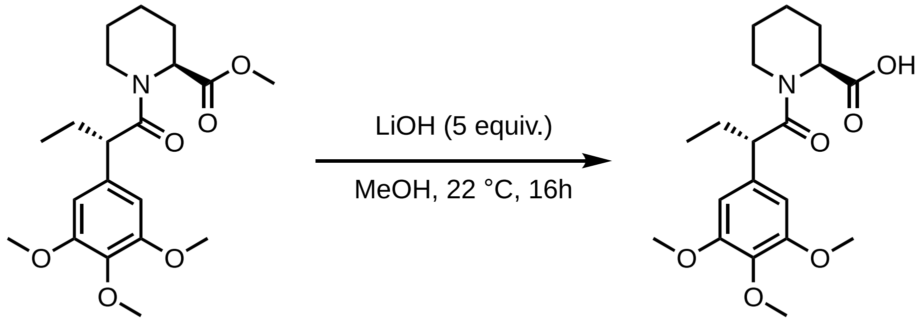

SI-6 **SI-7**

(*S*)-1-((*S*)-2-(3,4,5-Trimethoxyphenyl)butanoyl)piperidine-2-carboxylic acid (SI-7). In a 20 mL vial with a stir bar, **SI-6** (681.5 mg, 1.80 mmol, 1.0 equiv.) was dissolved in MeOH (3.6 mL) and LiOH (215.1 mg, 8.98 mmol, 5.0 equiv.) was added. The reaction stirred for 16 hours at 22 °C. 2N HCl was added until a white precipitate persisted. The aqueous mixture was extracted three times with EtOAc. The combined organics were washed with water and brine, dried with $MgSO_4$, filtered and concentrated. The crude residue was purified by preparative HPLC to give **SI-7** (416.6 mg, 1.14 mmol, 63.5% yield) as a white solid.

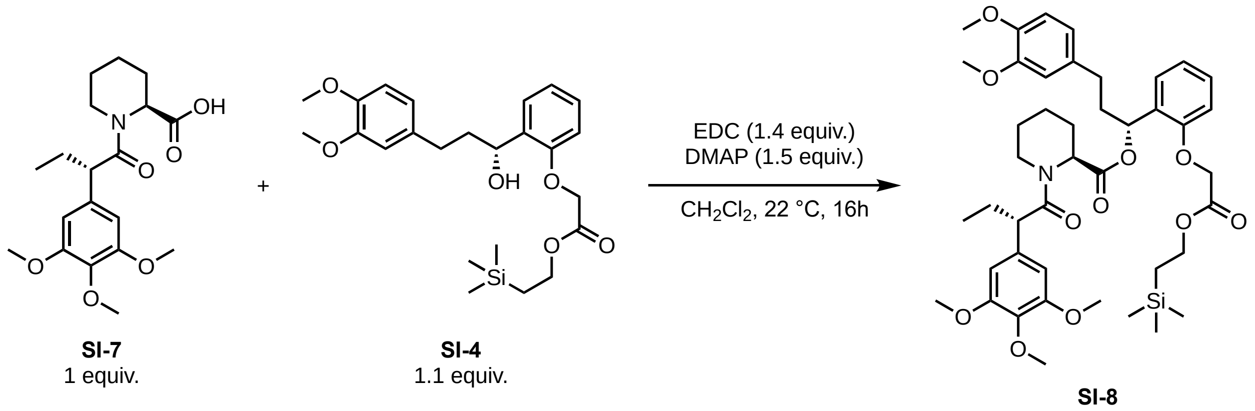

(*R*)-3-(3,4-Dimethoxyphenyl)-1-(2-(2-oxo-2-(2-(trimethylsilyl)ethoxy)ethoxy)phenyl)propyl (*S*)-1-((*S*)-2-(3,4,5-trimethoxyphenyl)butanoyl)piperidine-2-carboxylate (SI-8). In a 40 mL vial, **SI-7** (290.1 mg, 0.79 mmol, 1.0 equiv), **SI-4** (390.0 mg, 0.87 mmol, 1.1 equiv.), EDC (213.1 mg, 1.11 mmol, 1.4 equiv.), and DMAP (145.5 mg, 1.19 mmol, 1.500 equiv.) were dissolved in CH_2Cl_2 (7.9 mL). The reaction was stirred for 16 hours at 22 °C. The reaction was diluted with EtOAc and washed with 2N HCl, water, and brine. The organic layer was dried over $MgSO_4$, filtered, and concentrated. The crude mixture was purified by automated flash chromatography (RediSep Gold 80g, 0-100% EtOAc in hexane) to furnish **SI-8** (535.8 mg, 0.675 mmol, 85% yield) as a viscous, colorless oil.

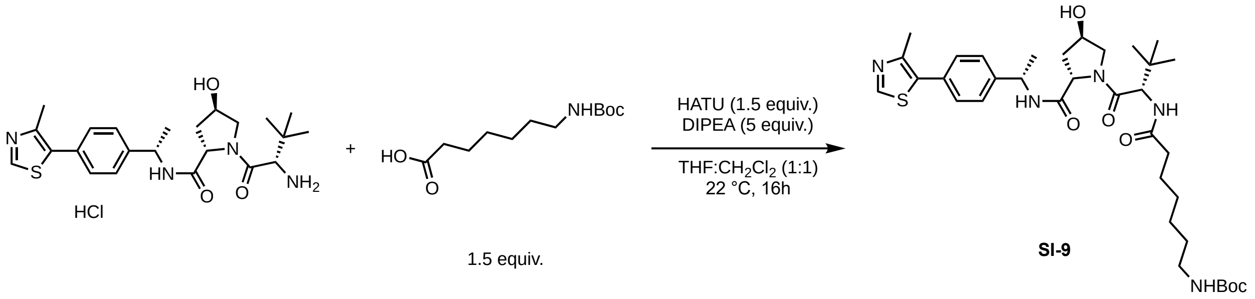

***tert*-Butyl (7-(((*S*)-1-((2*S*,4*R*)-4-hydroxy-2-(((*S*)-1-(4-(4-methylthiazol-5-yl)phenyl)ethyl)carbamoyl)pyrrolidin-1-yl)-3,3-dimethyl-1-oxobutan-2-yl)amino)-7-oxoheptyl)carbamate (SI-9)**. In a 2-dram vial, VHL ligand 2 hydrochloride (100.0 mg, 0.21 mmol, 1.0 equiv.), 7-((*tert*-butoxycarbonyl)amino)heptanoic acid (71.1 mg, 0.29 mmol, 1.4 equiv.), and HATU (110.2 mg, 0.29 mmol, 1.400 equiv.) were dissolved in a 1:1 mixture of CH_2Cl_2 (0.773 mL) and THF (0.773 mL). DIPEA (0.168 mL, 0.966 mmol, 4.6 eq) was added and the reaction stirred at 22 °C for 16 hours. The reaction was diluted with CH_2Cl_2 and washed with water and brine. The organics were dried over $MgSO_4$, filtered, and concentrated. The crude residue was purified by automated flash chromatography (RediSep Gold 24g, 0-15% CH_2Cl_2 in MeOH) yielded **SI-9** (114.8 mg, 0.171 mmol, 82% yield) as a colorless oil.

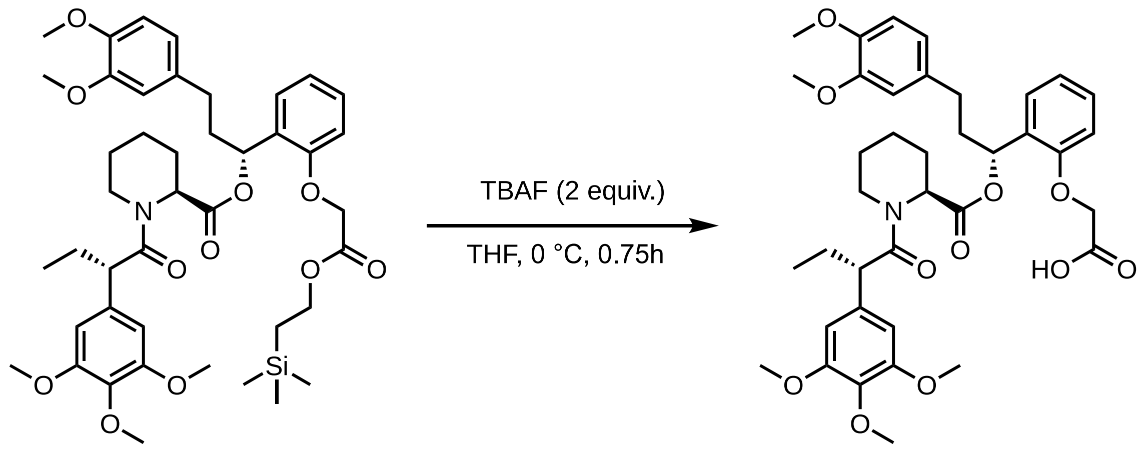

SI-8 **SI-10**

2-(2-((*R*)-3-(3,4-Dimethoxyphenyl)-1-(((*S*)-1-((*S*)-2-(3,4,5-trimethoxyphenyl)butanoyl)piperidine-2-carbonyl)oxy)propyl)phenoxy)acetic acid (SI-10). In a 2-dram vial, **SI-8** (195.0 mg, 0.25 mmol, 1.0 equiv.) was dissolved in THF (2.5 mL) and cooled in an ice bath. TBAF (1 M in THF, 0.491 mL, 0.491 mmol, 2.0 equiv.) was added and the reaction was monitored by LCMS. After 45 minutes LCMS showed clean conversion to **SI-10**. The reaction was concentrated by rotary evaporation and subjected to the next step immediately.

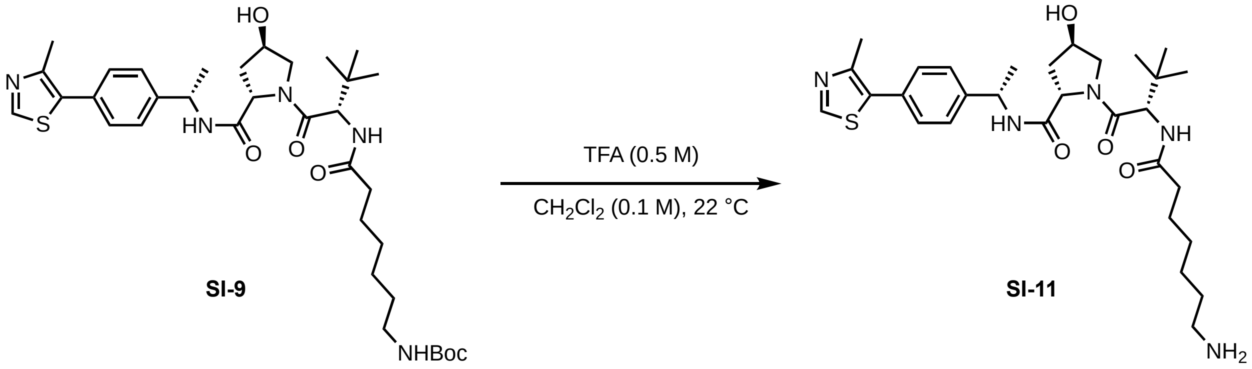

(2*S*,4*R*)-1-((*S*)-2-(7-Aminoheptanamido)-3,3-dimethylbutanoyl)-4-hydroxy-*N*-((*S*)-1-(4-(4-methylthiazol-5-yl)phenyl)ethyl)pyrrolidine-2-carboxamide (SI-11). In an 11.5 mL culture tube with a stir bar, **SI-9** (114.8 mg, 0.171 mmol, 1.0 equiv.) was dissolved in CH_2Cl_2 (1.7 mL). TFA (341 µL) was added and the reaction monitored by LCMS. Upon completion the reaction was quenched with saturated $NaHCO_3$ and extracted three times with CH_2Cl_2. The combined organics were dried over $MgSO_4$, filtered, and concentrated and **SI-11** was subjected to the next reaction without further purification.

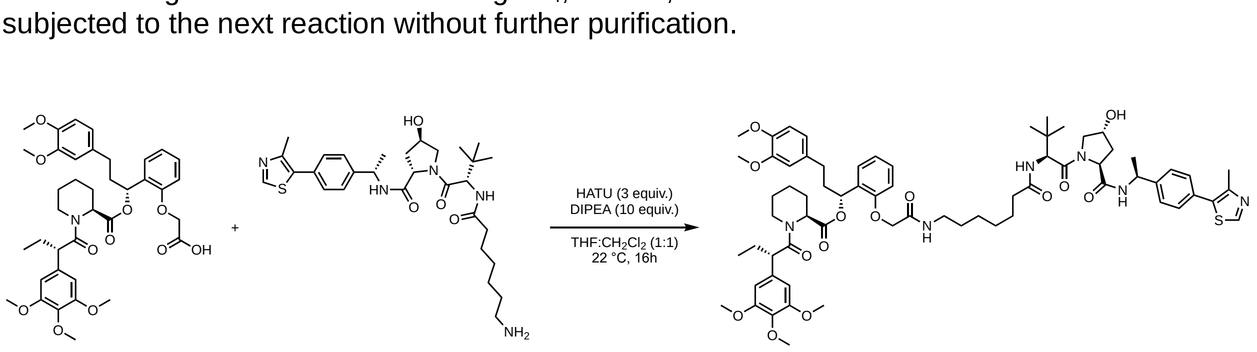

SI-10 1.5 equiv. **SI-11** 1 equiv. **dTAG-V1**

(*R*)-3-(3,4-Dimethoxyphenyl)-1-(2-(2-((7-(((*S*)-1-((2*S*,4*R*)-4-hydroxy-2-(((*S*)-1-(4-(4-methylthiazol-5-yl)phenyl)ethyl)carbamoyl)pyrrolidin-1-yl)-3,3-dimethyl-1-oxobutan-2-yl)amino)-7-oxoheptyl)amino)-2-oxoethoxy)phenyl)propyl (*S*)-1-((*S*)-2-(3,4,5-

trimethoxyphenyl)butanoyl)piperidine-2-carboxylate (dTAG-V1). In an 11.5ml culture tube with a stir bar, **SI-11** (97.7 mg, 0.171 mmol, 1.0 equiv.), **SI-10** (177.8 mg, 0.256 mmol, 1.5 equiv.), HATU (100 mg, 0.22 mmol, 1.5 equiv.) and DIPEA (0.150 mL, 0.86 mmol, 5.0 equiv.) were dissolved in a mixture of DCM (1.1 mL) and THF (1.1 mL). After 2 hours at room temperature, LCMS indicated product formation had stalled so additional HATU (100 mg, 0.22 mmol, 1.5 equiv.) and DIPEA (0.150 mL, 0.86 mmol, 5.0 equiv.) were added. The reaction stirred for an additional 14 hours at 22 °C. The reaction was concentrated and purified by automated flash chromatography (RediSep Gold 12g, 0-15% MeOH in CH_2Cl_2) furnished **dTAG-V1** (65.0 mg, 0.052 mmol, 31% yield) as a white solid.

^{1}H NMR (400 MHz, DMSO) δ 8.98 (s, 1H), 8.34 (d, *J* = 7.8 Hz, 1H), 7.81 – 7.65 (m, 2H), 7.46 – 7.36 (m, 4H), 7.25 – 7.16 (m, 1H), 6.88 – 6.71 (m, 4H), 6.66 – 6.58 (m, 2H), 6.56 (s, 1H), 6.29 – 5.96 (m, 1H), 5.37 – 5.27 (m, 1H), 5.11 – 5.02 (m, 1H), 4.97 – 4.87 (m, 1H), 4.60 – 4.46 (m, 3H), 4.42 (t, *J* = 7.7 Hz, 1H), 4.31 – 4.24 (m, 1H), 4.09 – 4.01 (m, 1H), 3.86 (t, *J* = 7.3 Hz, 1H), 3.76 – 3.68 (m, 9H), 3.65 – 3.58 (m, 3H), 3.58 – 3.54 (m, 6H), 3.16 – 2.98 (m, 2H), 2.45 (s, 3H), 2.25 – 1.85 (m, 8H), 1.85 – 1.74 (m, 1H), 1.66 – 1.50 (m, 4H), 1.50 – 1.28 (m, 9H), 1.27 – 1.13 (m, 6H), 0.93 (s, 9H), 0.87 – 0.76 (m, 3H).

Supplementary References

1. Nabet, B. et al. Rapid and direct control of target protein levels with VHL-recruiting dTAG molecules. Nat. Commun. 11, 4687 (2020).
2. Shimizu, M. et al. Metabolic profiles of pomalidomide in human plasma simulated with pharmacokinetic data in control and humanized-liver mice. Xenobiotica 47, 844–848 (2017).
3. Mosure, K. W. et al. Preclinical Pharmacokinetics and In Vitro Metabolism of Asunaprevir (BMS-650032), a Potent Hepatitis C Virus NS3 Protease Inhibitor. J. Pharm. Sci. 104, 2813–2823 (2015).
4. Sreekanth, V. et al. Chemogenetic System Demonstrates That Cas9 Longevity Impacts Genome Editing Outcomes. ACS Cent Sci 6, 2228–2237 (2020).

Source Data - Blot data for Supplementary Figures

Supplementary Figure 2a

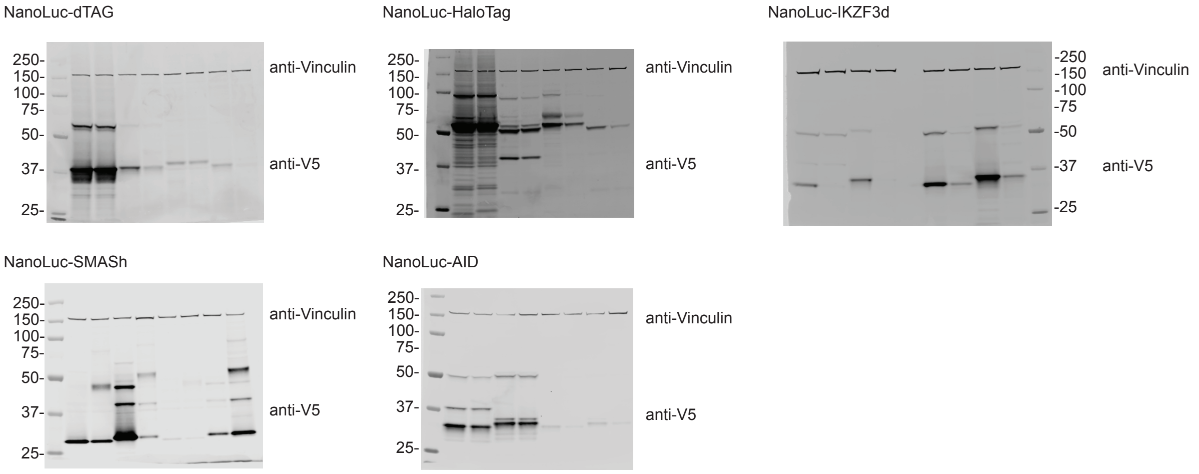

Supplementary Figure 2b

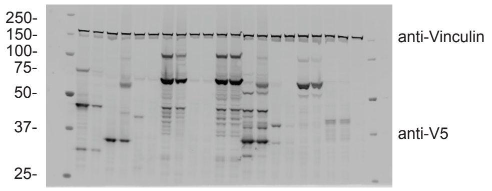

Supplementary Figure 3a

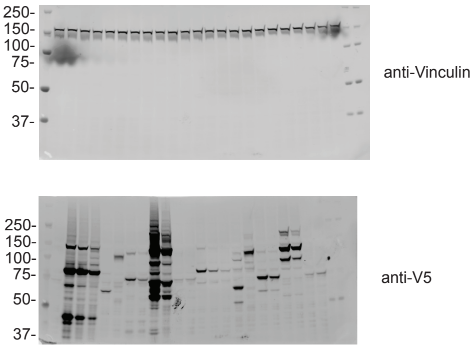

Supplementary Figure 3b

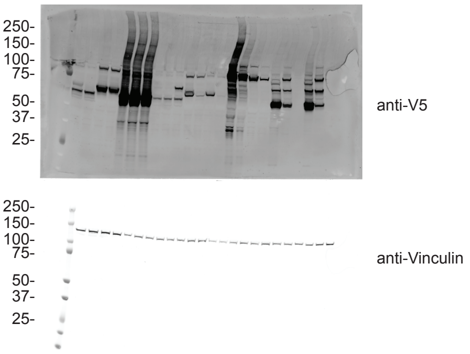

Supplementary Figure 3c

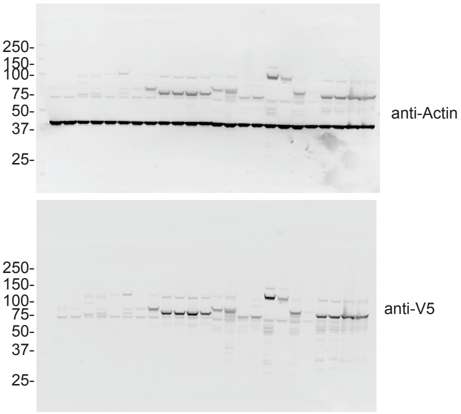

Supplementary Figure 4a

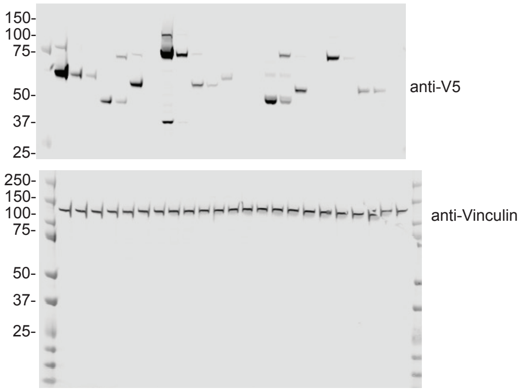

Supplementary Figure 4b (left)

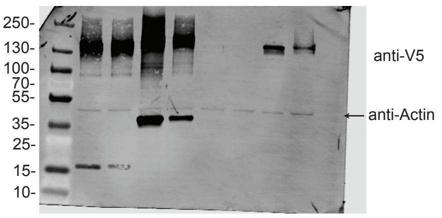

Supplementary Figure 4b (right)

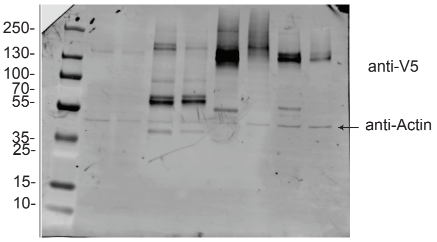

Supplementary Figure 4c

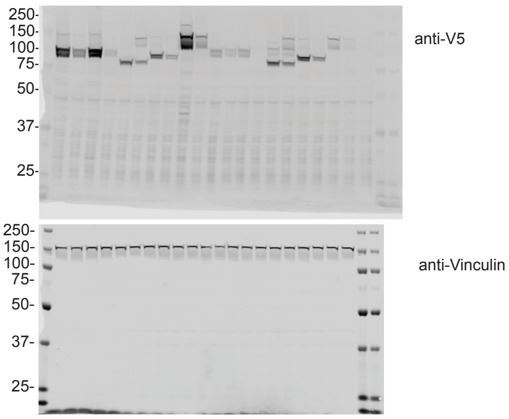

Supplementary Figure 4d

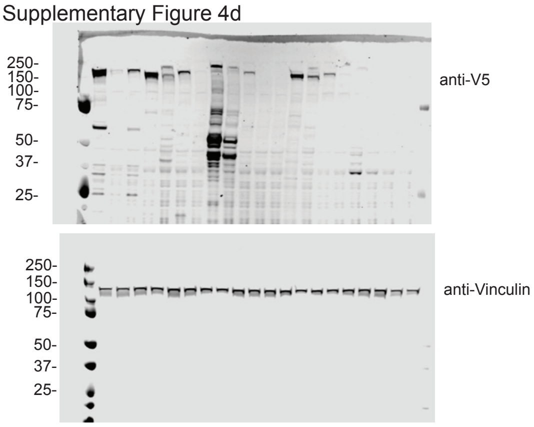

Supplementary Figure 5a

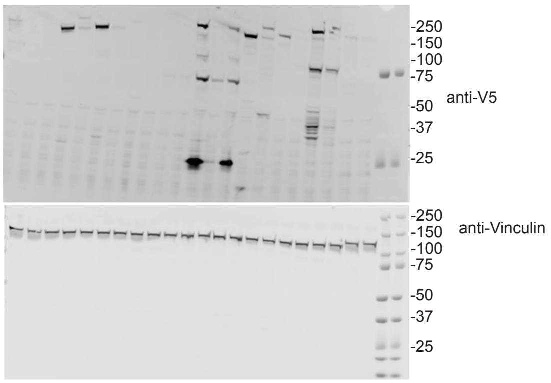

Supplementary Figure 5b

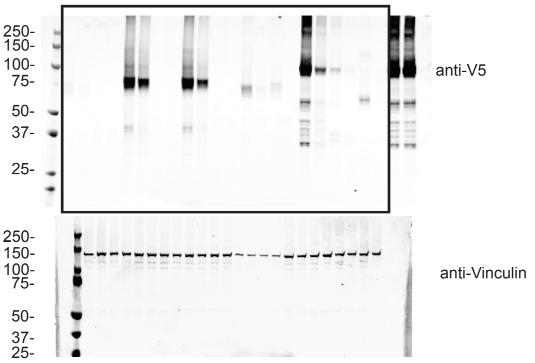

Supplementary Figure 5c

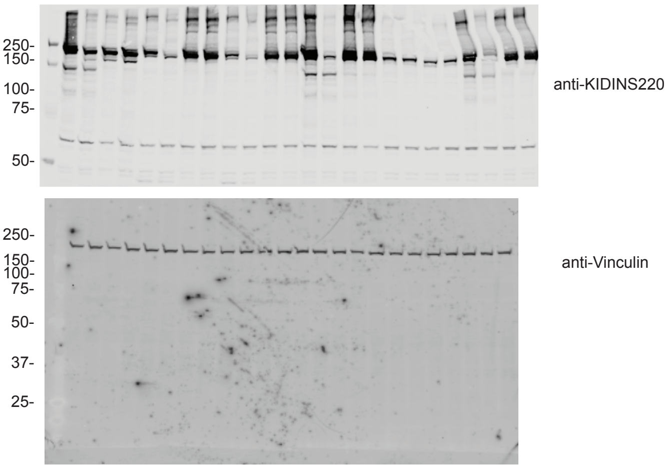

Supplementary Figure 5d

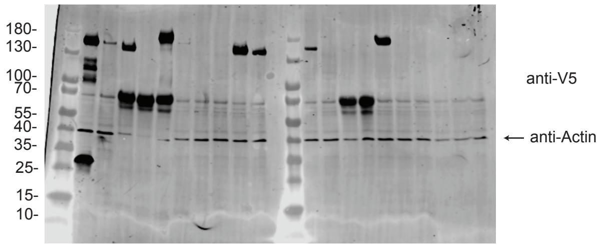

Supplementary Figure 6a

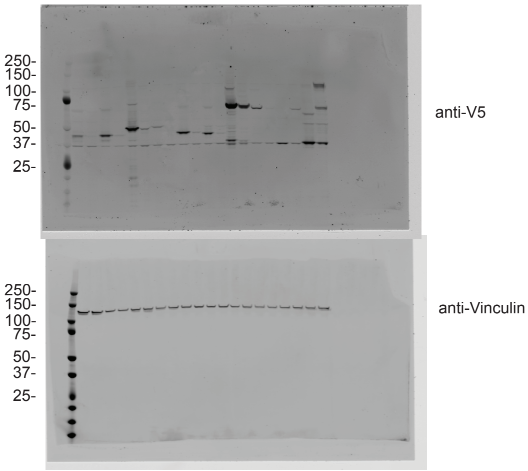

Supplementary Figure 6b

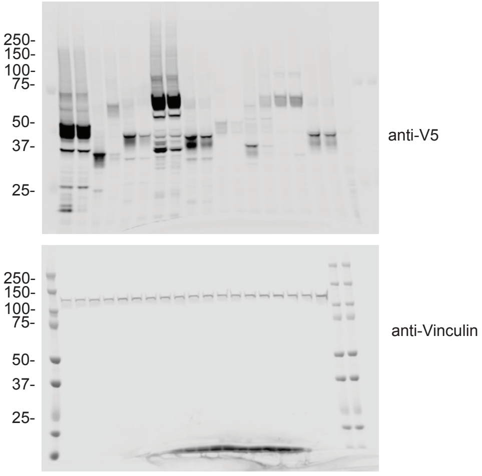

Supplementary Figure 6c

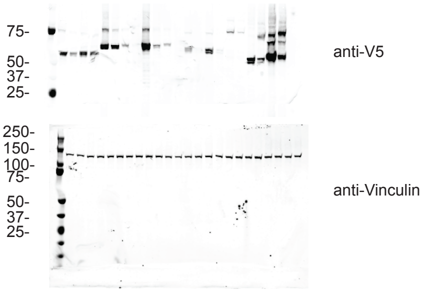

Supplementary Figure 6d

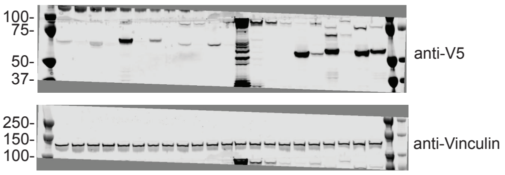

Supplementary Figure 7b

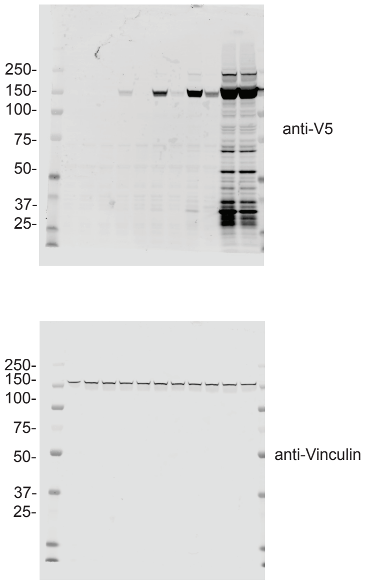

Supplementary Figure 7d

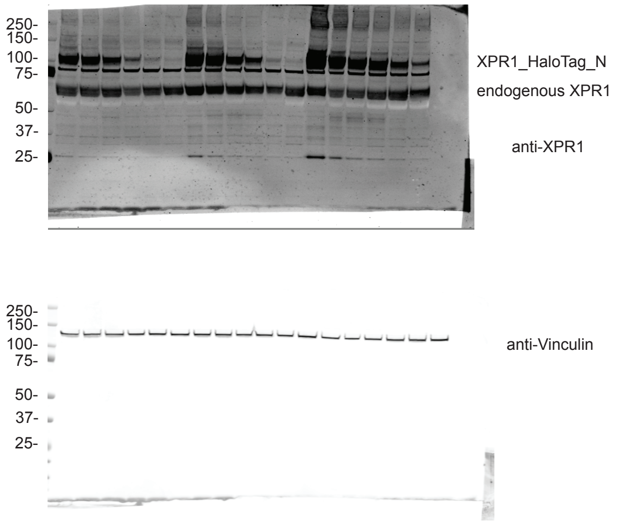

Supplementary Figure 8a - top

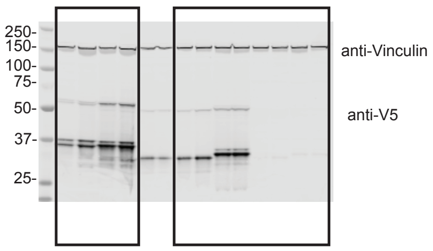

Supplementary Figure 8a - bottom

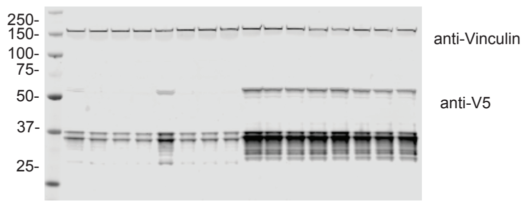

Supplementary Figure 8b

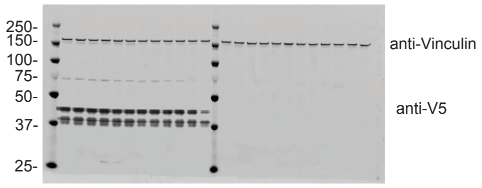

Supplementary Figure 8c

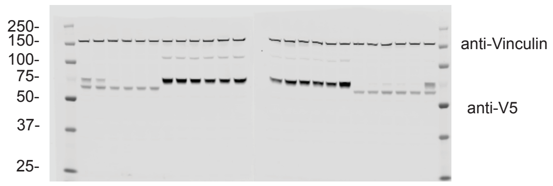

Supplementary Figure 8d

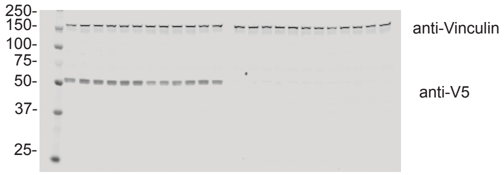

Supplementary Figure 8e

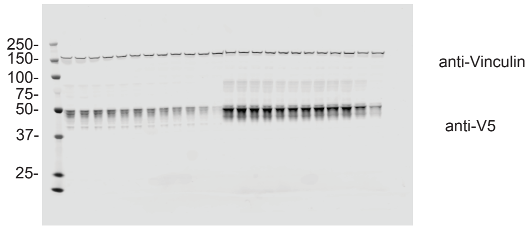

Supplementary Figure 8f

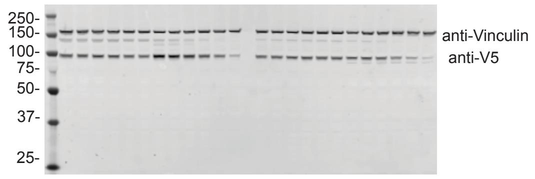

Supplementary Figure 8g

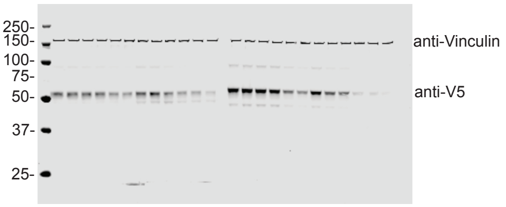

Supplementary Figure 8h

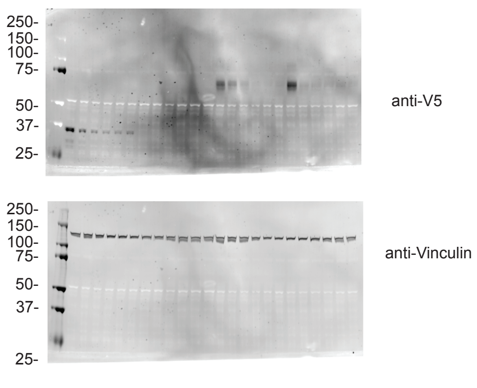

Supplementary Figure 8i

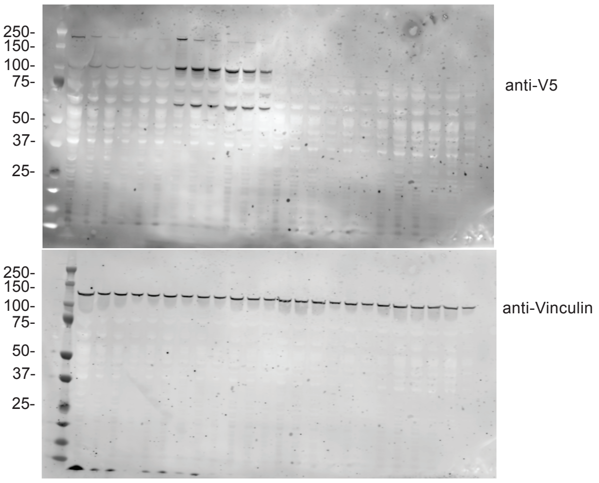

Supplementary Figure 9b

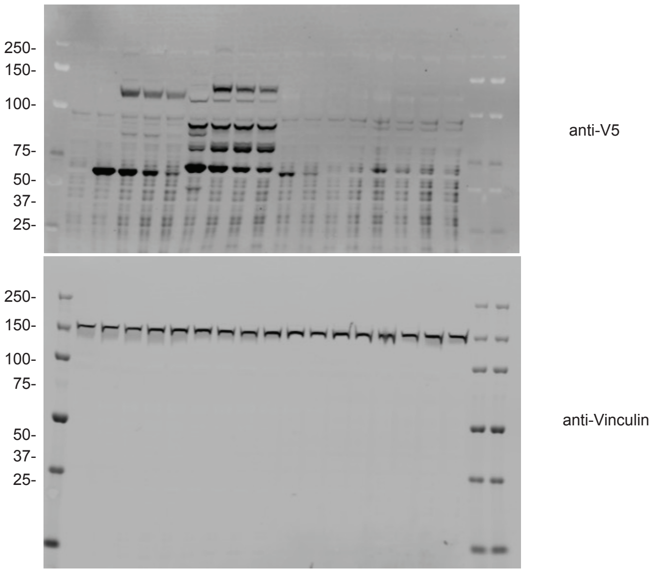

Supplementary Figure 9c

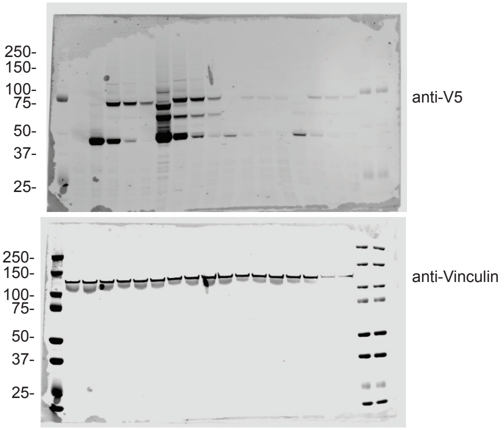

Supplementary Figure 9d

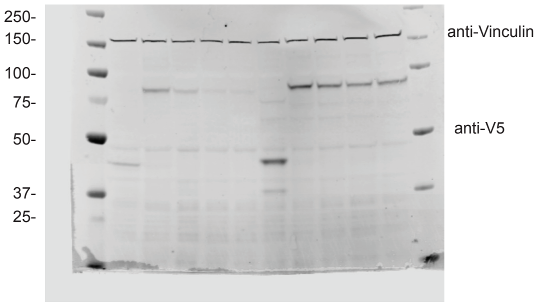

Supplementary Figure 10a

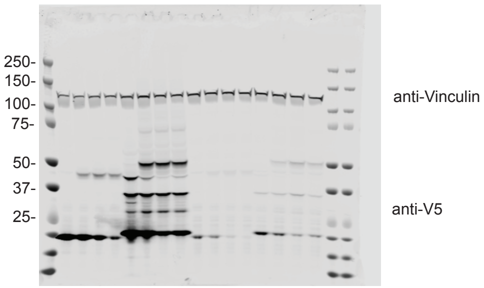

Supplementary Figure 10b

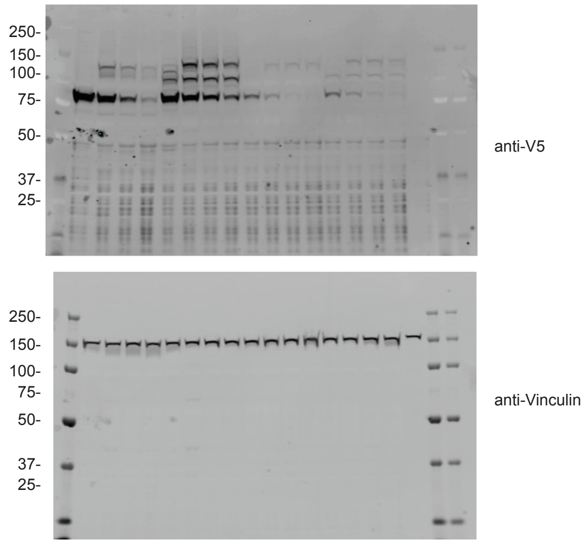

Supplementary Figure 10c

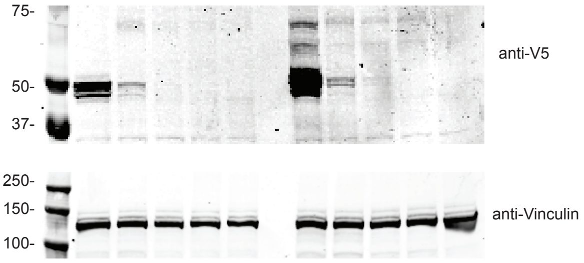

Supplementary Figure 10d

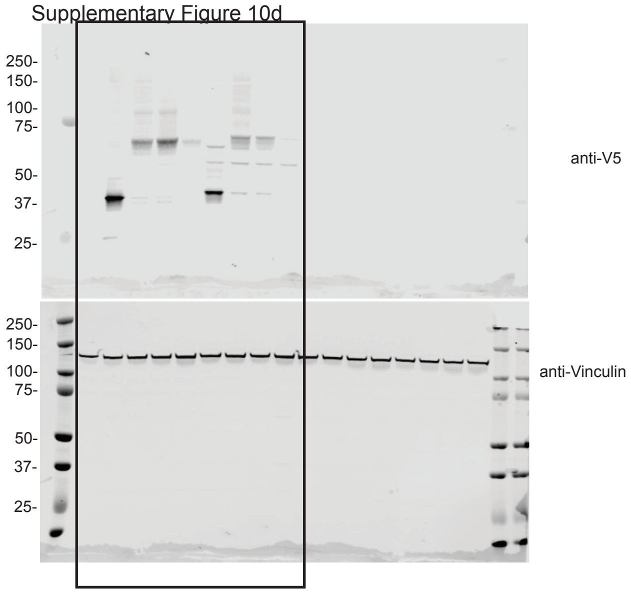

Supplementary Figure 10e

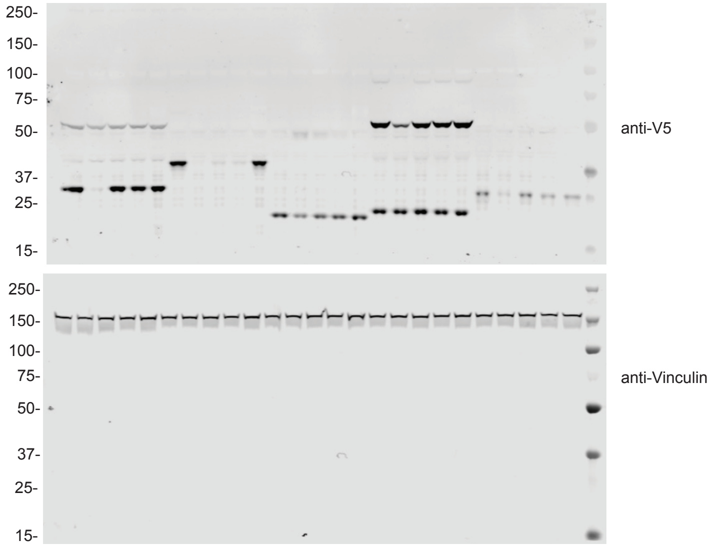

Supplementary Figure 10f

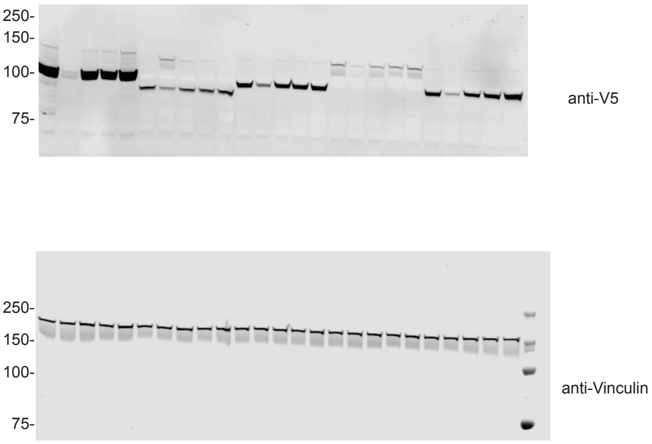

Supplementary Figure 11b

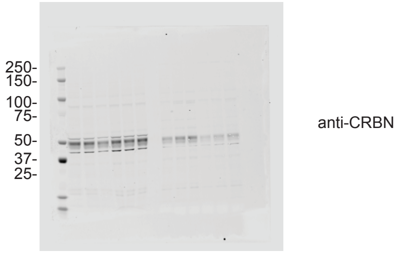

IGROV1 panel

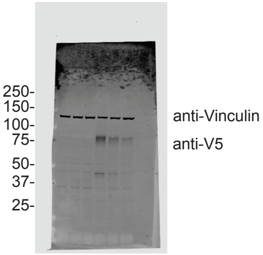

SNGM panel

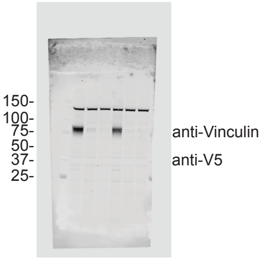

Supplementary Figure 11c

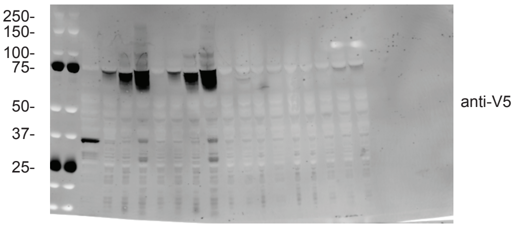

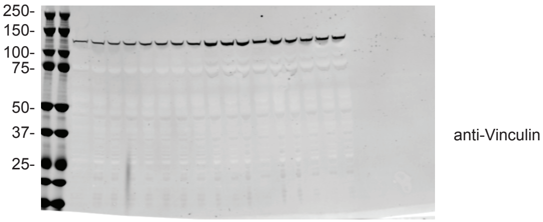

Supplementary Figure 11d

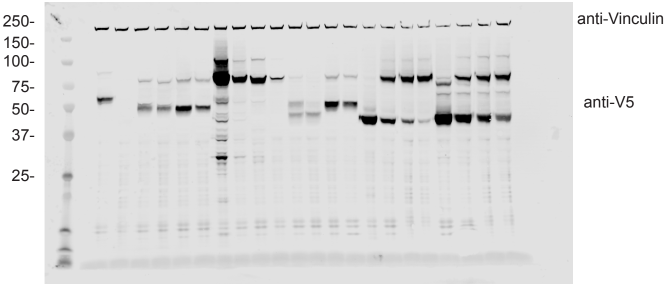

Supplement: Supplementary file 1 — Supplementary Information [file 41467_2022_33246_MOESM1_ESM.pdf]
