# Supplementary material for: Systematic profiling of conditional degron tag technologies for target validation studies
Source: Nat Commun. 2022 Sep 20;13:5495. doi: 10.1038/s41467-022-33246-4 (PMC9489723; doi:10.1038/s41467-022-33246-4)
Supplement: Supplementary file 3 — Reporting Summary [file 41467_2022_33246_MOESM3_ESM.pdf]

nature portfolio

Corresponding author(s): Alessandra Ianari

Last updated by author(s): Aug 3, 2022

Reporting Summary

Nature Portfolio wishes to improve the reproducibility of the work that we publish. This form provides structure for consistency and transparency in reporting. For further information on Nature Portfolio policies, see our Editorial Policies and the Editorial Policy Checklist.

Statistics

For all statistical analyses, confirm that the following items are present in the figure legend, table legend, main text, or Methods section.

n/a	Confirmed	
☐	☒	The exact sample size (*n*) for each experimental group/condition, given as a discrete number and unit of measurement
☐	☒	A statement on whether measurements were taken from distinct samples or whether the same sample was measured repeatedly
☐	☒	The statistical test(s) used AND whether they are one- or two-sided<br>*Only common tests should be described solely by name; describe more complex techniques in the Methods section.*
☒	☐	A description of all covariates tested
☒	☐	A description of any assumptions or corrections, such as tests of normality and adjustment for multiple comparisons
☐	☒	A full description of the statistical parameters including central tendency (e.g. means) or other basic estimates (e.g. regression coefficient) AND variation (e.g. standard deviation) or associated estimates of uncertainty (e.g. confidence intervals)
☐	☒	For null hypothesis testing, the test statistic (e.g. *F*, *t*, *r*) with confidence intervals, effect sizes, degrees of freedom and *P* value noted<br>*Give P values as exact values whenever suitable.*
☒	☐	For Bayesian analysis, information on the choice of priors and Markov chain Monte Carlo settings
☒	☐	For hierarchical and complex designs, identification of the appropriate level for tests and full reporting of outcomes
☒	☐	Estimates of effect sizes (e.g. Cohen's *d*, Pearson's *r*), indicating how they were calculated

Our web collection on statistics for biologists contains articles on many of the points above.

Software and code

Policy information about availability of computer code

Data collection	ImageStudio (v5.2) was used to collect immunoblot data.
Data analysis	GraphPad PRISM (v9.3.1); ImageStudio (v5.2), the R (v4.0.4) packages RStatix (v. 0.7.0), ggplot2 (v. 3.3.3), the program FlowJo (v. 10.8.1)

For manuscripts utilizing custom algorithms or software that are central to the research but not yet described in published literature, software must be made available to editors and reviewers. We strongly encourage code deposition in a community repository (e.g. GitHub). See the Nature Portfolio guidelines for submitting code & software for further information.

Data

Policy information about availability of data

All manuscripts must include a data availability statement. This statement should provide the following information, where applicable:

- Accession codes, unique identifiers, or web links for publicly available datasets
- A description of any restrictions on data availability
- For clinical datasets or third party data, please ensure that the statement adheres to our policy

All data is available within the manuscript and source data.

Field-specific reporting

Life sciences study design

All studies must disclose on these points even when the disclosure is negative.

Sample size	Due to the highly reproducible nature of the experiments performed in this manuscript, we typically evaluated technical duplicates and repeated each key finding at least 2 times with good concordance.
Data exclusions	No data was excluded.
Replication	All data is representative of at least two independent experiments.
Randomization	Randomization was not relevant to this study, since all of the work was done using cancer cell lines and groups chosen randomly would not have improved the robustness of the findings.
Blinding	Blinding was not relevant to this study, since the biases of the researcher were unlikely to influence the results of the reported experiments.

Reporting for specific materials, systems and methods

We require information from authors about some types of materials, experimental systems and methods used in many studies. Here, indicate whether each material, system or method listed is relevant to your study. If you are not sure if a list item applies to your research, read the appropriate section before selecting a response.

Materials & experimental systems

n/a	Involved in the study	
☐	☒	Antibodies
☐	☒	Eukaryotic cell lines
☒	☐	Palaeontology and archaeology
☒	☐	Animals and other organisms
☒	☐	Human research participants
☒	☐	Clinical data
☒	☐	Dual use research of concern

Methods

n/a	Involved in the study	
☒	☐	ChIP-seq
☐	☒	Flow cytometry
☒	☐	MRI-based neuroimaging

Antibodies

Antibodies used

V5 (Cell Signaling, D3H8Q)
In-cell western dilution of 1:250
Immunoblot dilution of 1:2,000

CellTag 800 (LI-COR, #926-41090)
In-cell western dilution of 1:500

Anti-Mouse 780 (LI-COR, #926-68070)
In-cell western dilution of 1:250
Immunoblot dilution of 1:10,000

Anti-Rabbit 800 (LI-COR, #926-32211)
Immunoblot dilution of 1:10,000

Vinculin (Sigma, V9131)
Immunoblot dilution of 1:5,00

KIDINS220 (Protein Tech, 21856-1-AP)
Immunoblot dilution of 1:2,000

XPR1 (Sigma , HPA016557)
Immunoblot dilution of 1:2,000

Anti-Mouse 488 (ThermoFisher, #A-11004)
Flow Cytometry dilution of 1:250

Validation

Anti-XPR1, Anti-KIDINS220, and Anti-V5 Antibodies were validated using lysates derived from cell lines that do not express the targets

Eukaryotic cell lines

Policy information about cell lines

Cell line source(s)	Each cell line was procured from ATCC: HEK-293T, IGROV1, MiaPACA2, HCT116, PK2, A375, and NIH-3T3.
Authentication	Routine STR profiling is performed on all cell lines to ensure their identity. Any cell line not matching the expected profile are discarded.
Mycoplasma contamination	All cell lines are routinely tested for Mycoplasma and discarded if found to be contaminated. No experiments in this manuscript were derived from cell lines known to be contaminated with Mycoplasma.
Commonly misidentified lines (See ICLAC register)	No commonly misidentified cell lines were used in this study.

Flow Cytometry

Plots

Confirm that:

- [x] The axis labels state the marker and fluorochrome used (e.g. CD4-FITC).
- [x] The axis scales are clearly visible. Include numbers along axes only for bottom left plot of group (a 'group' is an analysis of identical markers).
- [x] All plots are contour plots with outliers or pseudocolor plots.
- [x] A numerical value for number of cells or percentage (with statistics) is provided.

Methodology

Sample preparation	Cells were lifted from culture vessels using TrypLE Express (Thermo Fisher cat#12604013) and then diluted in PBS + 2% FBS. 300,000 cells were transferred to a U-bottom 96-well plate in 50 uL, followed by addition of 50 uL of XRBD staining solution. In Extended Data Figure 7c, the indicated doses were tested and in Figure 4d, XRBD was used at 100 nM. Cells were incubated at 37c for 40 minutes, washed once, and then incubated with an anti-mouse secondary antibody conjugated to AlexaFluor488 (ThermoFisher cat# A-11004). Cells were then incubated on ice for 40 minutes, washed four times in PBS + 2% FBS, and then analyzed on a CytoFlex LX instrument. At least 10,000 single cell events were recorded for each condition.
Instrument	CytoFlex LX
Software	Collection: CytExpert<br>Analysis: FlowJo
Cell population abundance	After sorting, at least 10,000 cells were analyzed.
Gating strategy	Excluded debris using FSC/SSC, and then selected for single cells using FSC-H v. FSC-A.

- [x] Tick this box to confirm that a figure exemplifying the gating strategy is provided in the Supplementary Information.
