# Supplementary figures and images for: Systematic profiling of conditional degron tag technologies for target validation studies
Source: Nat Commun. 2022 Sep 20;13:5495. doi: 10.1038/s41467-022-33246-4 (PMC9489723; doi:10.1038/s41467-022-33246-4)

Figure 4c

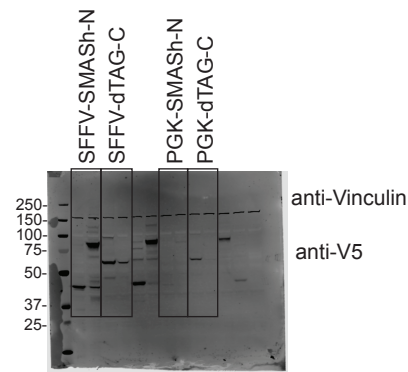

Supplement: Supplementary file 4 — Source Data [file 41467_2022_33246_MOESM4_ESM.zip › Source Data Files/6. Source Data - Blot Data.pdf]
